# Analyzing and modeling public interest in fishery resources: Proposing flagship species for promoting sustainable fisheries in Japan

**DOI:** 10.1371/journal.pone.0342833

**Published:** 2026-03-09

**Authors:** Shun Ota, Ken-ichi Hayashizaki

**Affiliations:** Marine Biosciences, Kitasato University, Sagamihara, Kanagawa, Japan; Central Marine Fisheries Research Institute, INDIA

## Abstract

Effective management of natural resources fundamentally relies on public support, making an understanding of the dynamics of public interest crucial for successful conservation policy. Globally, fisheries face sustainability challenges, yet public engagement often remains a key barrier to effective policy implementation. While public interest has traditionally been assessed through surveys, which are often costly and lack real-time granularity, digital trace data such as search engine queries offer a high-frequency alternative to monitor public attention. However, the primary drivers shaping public interest in specific fishery resources, and how these insights can be leveraged to select effective conservation symbols, remain poorly understood. Here we show, using Google Trends data for key Japanese fishery resources, that public interest is driven by two distinct archetypes—predictable seasonality and regional supply—and identify Pacific saury (*Cololabis saira*) as a unique species whose public profile is increasingly linked to its declining stock status. While previous assumptions might link consumer interest primarily to price and seasonal availability, our analysis reveals that for most species, market prices are a stronger driver than catch volumes. Crucially, Pacific saury diverges from this pattern; its public salience is uniquely influenced by both catch volume and a growing awareness of its resource depletion, making its profile more complex than that of a simple commodity. These findings demonstrate that the flagship species concept, traditionally applied to terrestrial megafauna, can be empirically adapted to exploited marine resources, providing a data-driven framework for selecting species that act as effective anchors for conservation messaging. Our methodology offers a low-cost, transferable workflow for integrating social data into resource management, a critical step for bridging the science-policy-society gap. By transforming passive digital footprints into actionable insights, this approach empowers conservation efforts to become more dynamic and responsive to public sentiment, ultimately fostering greater societal engagement in sustainability.

## 1 Introduction

Human societies derive numerous benefits from the diverse ecosystems on Earth, including food provision and climate regulation, which are widely recognized as “ecosystem services” [1]. Among these, fishery products, including various fish species, represent a crucial component of ecosystem services that directly impact daily life and play an indispensable role in global food security [2,3]. Particularly in coastal regions, fisheries contribute not only to economic benefits but also to cultural and traditional values, serving as essential elements for social and economic stability. Therefore, the sustainable management and promotion of fisheries are not only necessary for ensuring food production but also for maintaining local communities and preserving cultural heritage [4,5].

However, the marine environment is among the most exploited ecosystems worldwide [6,7]. The depletion of commercially and economically significant fishery resources has become a critical global issue [8]. According to the Food and Agriculture Organization of the United Nations (FAO), approximately 38% of the world's fishery resources are currently overexploited [9]. In response to growing concerns regarding ecosystem functions and services, international efforts to conserve and sustain these resources have gained significant attention [2,10]. Against this backdrop, public attention to sustainable resource use is highly heterogeneous across regions, and socio-cultural evidence remains limited [11,12].

Although global fisheries production has increased in recent years due to the expansion of aquaculture, Japan’s fisheries production has declined significantly over the past few decades. This decline is largely attributed to resource depletion [13–15]. Additionally, consumer awareness and interest in sustainable fisheries and certification systems, such as eco-labeling, remain low in Japan [16–19], posing a challenge to the realization of sustainable fisheries [20,21]. Moreover, despite large-scale reforms in fisheries resource conservation policies, insufficient monitoring systems and enforcement mechanisms have hindered their effectiveness [22]. Similar to challenges observed in climate change legislation, where limited public interest and understanding have been shown to impede policy uptake [23], the lack of consumer understanding regarding sustainable fisheries may also play a critical role in preventing broader societal adoption.

Beyond fisheries, numerous conservation projects and ecosystem management initiatives have demonstrated that public interest is a critical determinant of policy support and institutional success. Understanding and effectively managing this public attention is therefore essential for ensuring the legitimacy, effectiveness, and long-term sustainability of conservation policies [24]. Japan provides a particularly salient case: although it remains one of the world’s major fishing nations, governance of living marine resources has been criticized as lagging behind other developed countries [25,26]. Amid declining landings and stock concerns, public attention to sustainable fisheries in Japan remains limited, offering a realistic setting to examine how low-cost attention signals can inform the timing, targeting, and species choice of outreach.

Such challenges are widely recognized in the field of conservation ecology, where the concept of “flagship species” has been employed as a potential solution. A flagship species is a particular organism that serves as a symbolic representation of conservation efforts, attracting public interest and driving successful conservation initiatives. This concept was originally defined by Simberloff [27] as “a conspicuous single species that functions as a symbol for an entire ecosystem and becomes the focal point of conservation campaigns.” Traditional flagship species were typically charismatic animals, such as tigers, elephants, and pandas, that served as icons for conservation efforts [28]. However, previous studies have suggested that the selection of flagship species should consider not only global charisma but also local cultural contexts [29].

More recently, the flagship species concept has evolved beyond serving merely as ecological surrogates, with an increasing emphasis on their role as “strategic conservation tools” [30–33]. These researchers highlight how flagship species can attract stakeholders and public interest, generate support, and integrate with political and economic factors to enhance the success of conservation efforts.

Despite its widespread use in conservation ecology, the adoption of flagship species in promoting sustainable fisheries remains largely unexplored. Given that fisheries represent both exploited resources and essential components of cultural and food systems, this absence constitutes a significant gap in linking public interest with sustainable resource use.

This study therefore confronts a central challenge at the intersection of conservation, marketing, and governance: can we identify a species that transcends simple market dynamics to serve as a powerful symbol for an entire industry in crisis? To address this question, we analyze public interest in key Japanese fishery resources to identify its underlying drivers, ultimately proposing a data-driven framework for selecting flagship species. By using digital trace data from Google Trends, our approach provides a transferable methodology that offers novel insights not only for Japan, but also for fisheries governance worldwide, where integrating public interest into management has often been overlooked.

## 2 Materials and methods

### 2.1 Google Trends

Google Trends (https://trends.google.com/trends/) [34] is a freely accessible online database that provides time-series data on search volumes for specific keywords entered into Google's search engine. Introduced by Google in 2006, this database has been widely utilized as a research tool across various disciplines, including marketing, economics, public health, and social sciences [35]. Notably, Google Trends is a valuable method for understanding the dynamics of public interest, with applications extending beyond consumer behavior and market trends to include forecasting infectious disease outbreaks and assessing political interest [36,37].

Note that Google Trends is based on an anonymized, categorized, and aggregated sample of actual Google searches. It removes repeated searches and excludes queries generated by Google products and services, including internal searches from AI Mode and AI Overviews. Therefore, AI-generated queries within Google’s own search products are unlikely to directly affect the Trends series. In addition, because the Google Trends time series analyzed in this study covers January 2010 through December 2023, potential distortions from generative-AI search interfaces are unlikely to affect the period analyzed.

In recent years, Google Trends has also been increasingly employed in the fields of biodiversity and environmental conservation [38,39]. For example, Jarić et al. [40,41] used Google Trends to demonstrate that public interest in biodiversity conservation can change rapidly over short periods. Their findings suggest that Google Trends can serve as an effective tool for continuously monitoring consumer awareness in environmental issues.

Prior work has mapped public perceptions of marine issues and the science–policy–society interface largely through cross-national surveys and media-based corpora, but behavior-based, comparable evidence remains limited. Traditional surveys and official statistics are indispensable, yet they are not designed to capture real-time fluctuations in public attention. In contrast, aggregated search behavior offers a complementary, high-frequency indicator that can be processed at scale. Accordingly, we use Google Trends search volume as a proxy for public attention to fishery resources, and we pair it with statistical series as described below.

### 2.2 Data collection

In this study, search volume data obtained from Google Trends were used as an indicator to measure public interest in the selected fish species. All data used in this study were collected in May 2024. The analysis focused on fish species subject to Total Allowable Catch (TAC) regulations in Japan as of 2024, excluding Pacific bluefin tuna (*Thunnus thynnus*). The targeted species included Japanese horse mackerel (*Trachurus japonicus*), Japanese sardine (*Sardinops melanostictus*), mackerel species (chub mackerel: *Scomber japonicus* and spotted mackerel: *Scomber australasicus*), Pacific saury (*Cololabis saira*), Alaska pollock (*Gadus chalcogrammus*), Japanese flying squid (*Todarodes pacificus*), and snow crab (*Chionoecetes opilio*).

Pacific bluefin tuna was excluded from the analysis due to the difficulty in accurately identifying search terms, as the term “Maguro” (tuna) is commonly used to refer to multiple tuna species, making it challenging to isolate searches specifically related to *Thunnus thynnus*. The keyword selection process is illustrated below, using Pacific saury as an example.

(1) Listing Common Names
For each fish species, commonly used names were identified, including their hiragana, katakana, and kanji representations, by referencing sources such as Wikipedia and online dictionaries. For example, in the case of Pacific saury, the terms “さんま (Hiragana),” “サンマ (Katakana)” and “秋刀魚 (Kanji)” were considered.
(2) Comparison Using Google Trends
Search volumes for all candidate terms were compared to determine the most frequently searched keyword. This comparison was based on the average relative search volume over the entire study period (2010–2023). In cases where average values were close, the term with the highest peak search volume was selected.
(3) Validation of Search Results
A manual Google search was conducted to verify whether the selected keyword primarily returned information relevant to the target fish species. If unrelated results appeared frequently, the next most searched keyword was considered instead.

For instance, in the case of Pacific saury, the term “Sanma (Hiragana)” was found to generate numerous search results related to a person’s name rather than the fish. As a result, “Sanma (Katakana)” the second most searched term, was adopted as the appropriate keyword. Similar ambiguities were addressed for other species. For example, for Japanese sardine, the search term “Iwashi (Hiragana)” was compared against “Katakuchi-Iwashi” and “Urume-Iwashi,” and since the latter two had negligible search volumes, “Iwashi (Hiragana)” was selected. In the case of Alaska pollock, searches for “Tara (Katakana)” and “Tara (Hiragana)” were found to overlap significantly with “Madara (Pacific cod),” leading to the adoption of “Sukesoudara (Katakana)” as the optimal keyword.

The final keywords selected for each species were as follows:

Japanese horse mackerel: “Aji (Hiragana)”
Japanese sardine: “Iwashi (Hiragana)”
Mackerel species: “Saba (Katakana)”
Pacific saury: “Sanma (Katakana)”
Alaska pollock: “Sukesoudara (Katakana)”
Japanese flying squid: “Surumeika (Katakana)”
Snow crab: “Zuwaigani (Katakana)”

The monthly relative search volume (RSV) data for each keyword was obtained from Google Trends, covering the period from January 2010 to December 2023. Google Trends reports relative search volume (RSV), a normalized index scaled from 0 to 100 within the specified time window and region rather than absolute search counts. Data collection was configured to the “Japan” region, “all categories,” and “web search” only. Additionally, the related search queries and prefecture-level search volumes were collected as annual data from 2010 to 2022.

Furthermore, supplementary datasets were obtained for comparative analysis: monthly catch volumes were obtained from the market-distribution statistics portal of the Fisheries Agency of Japan (https://www.market.jafic.or.jp/fKoukaiMain.html) [42]. Consumer Price Index (CPI) data (excluding Alaska pollock and snow crab) was obtained from the Statistics Bureau of Japan (https://www.stat.go.jp/data/cpi/index.htm) [43]. Market transaction prices from the Tokyo Metropolitan Central Wholesale Market were obtained as monthly data from the Tokyo Metropolitan Government website (https://www.shijou.metro.tokyo.lg.jp/torihiki/geppo) [44]. Prefecture-level annual catch volumes were obtained from Ministry of Agriculture, Forestry and Fisheries website (https://www.maff.go.jp/j/tokei/kouhyou/kaimen_gyosei/index.html) [45]. All datasets were collected and analyzed in accordance with the terms of service and usage policies of each data provider. For analysis, all datasets were integrated by species into a single time-series file, and the same file also includes values derived during preprocessing and statistical analyses. In addition, the Tokyo Metropolitan Government has changed the public dissemination format for the Central Wholesale Market statistics, and some historical pages are no longer directly accessible. To ensure reproducibility, we retained the processed monthly price series used in this study as part of the analysis dataset.

### 2.3 Statistical analysis

All statistical analyses in this study were performed using the SciPy 1.15.1 statistical analysis library in Python 3.12.2, while figures and tables were generated using the Matplotlib 3.8.3 visualization library. To ensure methodological consistency with the Consumer Price Index (CPI), the market price data was indexed by setting the 2023 average price to a value of 100. Subsequently, all time-series datasets were standardized using a z-score transformation (mean of 0 and standard deviation of 1).

#### 2.3.1 Seasonal-trend decomposition using LOESS.

To evaluate the relationships between the time-series data, all datasets were first standardized and then decomposed using Seasonal-Trend decomposition using LOESS (STL). Given the monthly nature of the data, the seasonal period was explicitly set to 12. To enhance the robustness of the decomposition against outliers, the smoothing parameter for the seasonal component was set to seasonal = 13. This process allowed for the extraction of trend, seasonal, and residual components from the original time-series data.

#### 2.3.2 Spearman's rank correlation coefficient.

Using the residual components obtained from STL decomposition and the detrended data (calculated as the original data minus the trend component), the correlation between search volume and fishery catch volume, as well as between search volume and the Consumer Price Index (CPI), was analyzed. The Jarque-Bera (JB) test confirmed that all fish species data, except for Japanese horse mackerel, followed a non-normal distribution. Therefore, Spearman's rank correlation coefficient was employed to evaluate correlations at a 5% significance level.

#### 2.3.3 Relationship between prefecture-level catch volumes and search volumes.

To understand the relationship between the regional distribution of public interest in each fish species and actual fishery catch volumes, an analysis was conducted using prefecture-level annual search volume data and catch volume data in 2022. The prefecture-level search volume is represented as a relative value, indicating the proportion of searches for a specific keyword relative to the total number of searches conducted in each prefecture. This metric is normalized such that the highest value is set to 100. For prefecture-level catch volume, considering the potential for high variability in values, a log10(x + 1) transformation was applied before analysis to mitigate skewness. As in the monthly analysis, given the potential non-normality of data distributions, Spearman’s rank correlation coefficient was used to evaluate the relationship between search volume and catch volume for each prefecture.

#### 2.3.4 Collection and analysis of related search keywords.

To examine the types of information sought when consumers search for specific fish species, related search keywords were collected from Google Trends as annual data from 2010 to 2023 nationwide in Japan. Google Trends provides a list of keywords frequently searched in association with a given keyword, allowing for an assessment of consumer interest beyond the primary search term. Google Trends generates related search keywords based on user behavior, reflecting commonly searched terms that co-occur with the target keyword. This approach enables the identification of trending topics and evolving search patterns in relation to each fish species. By analyzing related search keywords, this study aims to provide insights into consumer awareness and interest in fisheries-related topics, highlighting shifts in public concern and potential areas for targeted communication strategies in sustainable fisheries management.

#### 2.3.5 Path analysis.

**Fig 1:**
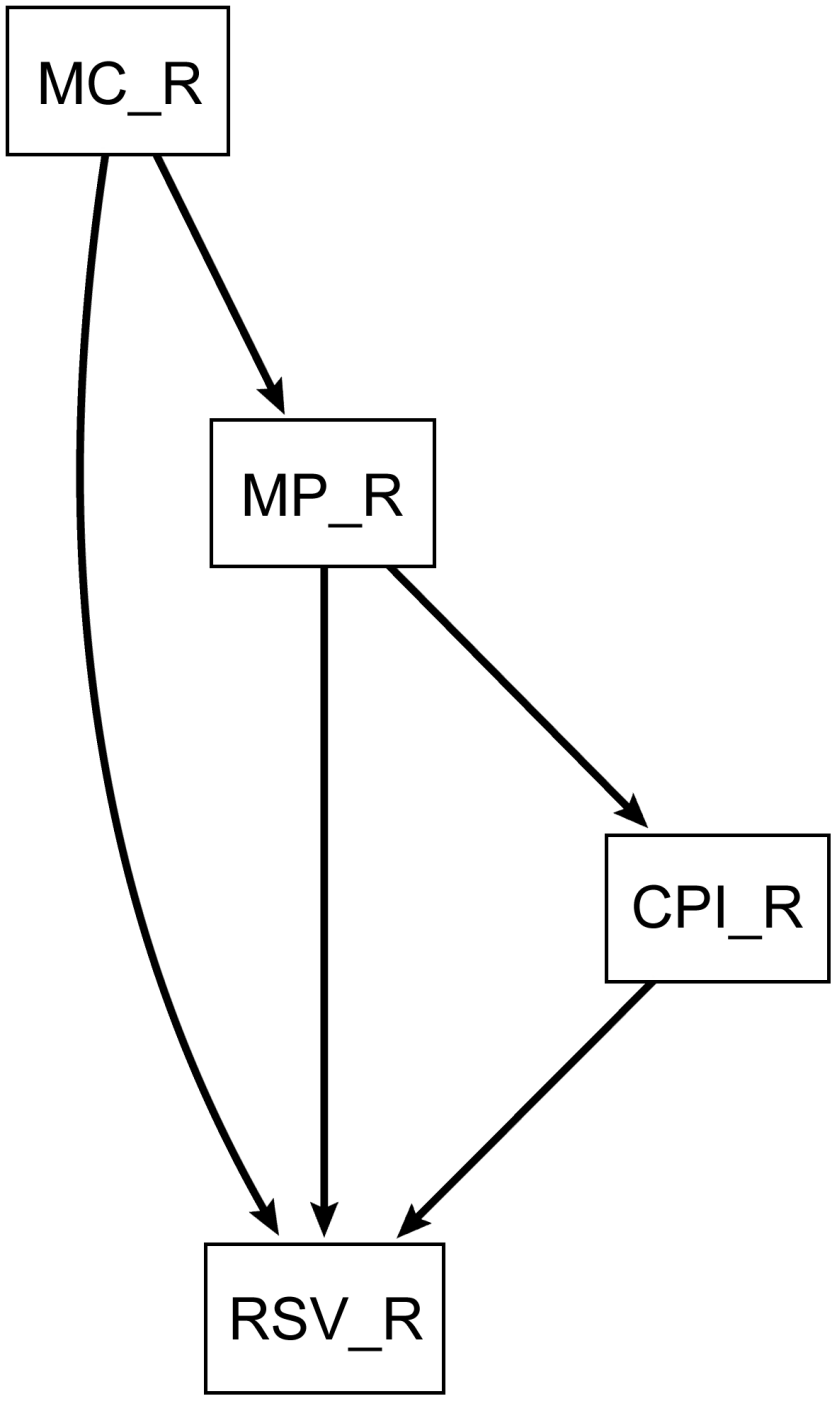
Conceptual path analysis illustrating the hypothesized relationships between variables. The model posits that fishery catch volume (MC_R) influences market price (MP_R), which in turn affects the Consumer Price Index (CPI_R). Public search volume (RSV_R) is influenced by all three preceding variables.

To elucidate the dynamics of public interest in the fish species targeted in this study and identify its driving factors, path analysis was employed. The time-series data used in the analysis were the residual components extracted through STL decomposition. The path analysis model was constructed based on theoretical causal relationships, taking into account the results of previous analyses (Fig 1).

Specifically, the model assumed the following relationships:

Market price (MP_R) is influenced by fishery catch volume (MC_R).
Consumer Price Index (CPI_R) is influenced by market price (MP_R).
Search volume (RSV_R) is influenced by fishery catch volume (MC_R), market price (MP_R), and consumer price index (CPI_R).

Each variable was defined as the residual component obtained through data preprocessing, and latent factor analysis was not performed. In the baseline specification, we did not estimate residual covariances; contemporaneous associations among predictors were represented by directed paths. As a robustness check, we additionally allowed a residual covariance between wholesale and retail prices (MP_R ~~ CPI_R) while retaining the pass-through path (CPI_R ~ MP_R); model fit and path estimates were virtually unchanged, so the covariance-free specification was adopted.

The analysis was conducted using the Semopy 2.3.11 library in Python. Estimation was performed using the maximum likelihood method (ML), with Sequential Least Squares Programming (SLSQP) employed as the optimization algorithm. After estimation, the model’s goodness of fit was evaluated using multiple fit indices, including the chi-square test, Comparative Fit Index (CFI), and Root Mean Square Error of Approximation (RMSEA), based on commonly accepted criteria (e.g., CFI > 0.95, RMSEA < 0.08). Additionally, variance inflation factors (VIF) were calculated to examine multicollinearity among variables and verify the model's stability. All VIF values were below 3.0, indicating that multicollinearity was not a significant issue in our model. Finally, path diagrams were initially generated using Graphviz 0.20.3 to determine the layout. The final figures were manually redrawn and formatted based on the Graphviz output; all coefficients and significance annotations were taken directly from the results.

## 3 Results

### 3.1 Seasonal-trend decomposition using LOESS

The results of the time-series analysis revealed that both fisheries catch volume and search volume exhibited seasonal periodicity across all fish species. Additionally, the fish species could be broadly categorized into two groups based on the similarity of their catch volume and search volume patterns:

1. Species with similar seasonal patterns in catch volume and search volume (Japanese horse mackerel and Pacific saury).
2. Species with differing seasonal patterns in catch volume and search volume (Japanese sardine, Japanese flying squid, snow crab, Alaska pollock, and mackerel species).

These findings indicate that the temporal relationship between fishery catch volume and public interest varies across species.

### 3.2 Temporal dynamics: Price and seasonality as key drivers of public interest

#### 3.2.1 Spearman’s rank correlation coefficient: Search volume and fishery catch volume.

The analysis revealed that species can be partitioned into those exhibiting seasonal synchrony between the annual peak in catch volume and the peak in search interest (hereafter, “Seasonal Fish”) and those without such synchrony (“Non-seasonal Fish”).

(i) Seasonal Fish
Japanese horse mackerel, Japanese sardine, and Pacific saury showed synchronization between the seasonal peak in catch volume and the peak in search volume (Figs S1.1-S1.3 in S1 File). This pattern was statistically supported by Spearman’s rank correlations (Figs S2.1-S2.3 in S1 File). In addition, significant positive correlations were detected in the residual component in specific months—August and December for horse mackerel (Fig S3.1 in S1 File), and September for Pacific saury (Fig S3.2 in S1 File), indicating episodic coupling beyond the general seasonal trend.
(ii) Non-seasonal Fish
The remaining species did not show synchronization between catch and search peaks (Figs S1.4-S1.7 in S1 File). Japanese flying squid and snow crab exhibited distinctive scatter patterns, consistent with months in which factors other than low catch (e.g., market or consumption-side drivers) increased search interest (Figs S2.4 and S2.5 in S1 File). Consistent with this lack of synchrony, both Alaska pollock and mackerel species showed no significant overall correlation between detrended catch and search volume (Figs S2.6 and S2.7 in S1 File). For Alaska pollock, significant negative residual correlations were observed in January and October.

#### 3.2.2 Spearman’s rank correlation coefficient: Search volume and consumer price index (CPI).

The analysis revealed that species can be grouped into three categories: Price-sensitive (higher search volume when CPI is lower), Price-positive (higher search volume when CPI is higher), and Price-neutral (no significant association).

(i) Price-sensitive
Japanese horse mackerel and Japanese sardine tended to be searched more frequently in months with lower CPI (Figs S4.1 and S4.2 in S1 File). In particular, Japanese sardine also showed a significant negative correlation in the residual component, suggesting that short-term price declines may heighten public interest in this species. Significant negative residual correlations were detected in May, October, and December for Japanese sardine (Fig S5.1 in S1 File), and in December for Japanese horse mackerel (Fig S5.2 in S1 File), implying that price reductions in specific months may trigger increases in search volume.
(ii) Price-positive
Pacific saury was searched more frequently in months with higher prices (Fig S4.3 in S1 File). This contrasts with Japanese horse mackerel and Japanese sardine, for which prices typically fall when catches increase. In September—when catch, demand, and supply rise simultaneously—the CPI often remains elevated, likely reflecting species-specific demand–supply conditions for Pacific saury (Fig S5.3 in S1 File). A strong negative residual correlation was also observed in December.
(iii) Price-neutral
Japanese flying squid and mackerel species showed no significant overall association between CPI and search volume (Figs S4.4 and S4.5 in S1 File). However, Japanese flying squid exhibited a negative correlation in the residual component, suggesting that temporary price declines may spur increases in search interest.

### 3.3 Spatial dynamics: Regional catches shape local interest

Similar to the time-series analysis, the relationship between prefecture-level catch volume and search volume varied across fish species. For Japanese horse mackerel and Japanese sardine, a weak positive correlation was observed (Figs S6.1 and S6.2 in S1 File). The results suggest that Japanese horse mackerel is more frequently searched in southern prefectures, whereas Japanese sardine is more frequently searched in northern prefectures. This implies that while both species are widely recognized as staple seafood products, consumer interest tends to be higher in regions where they are more frequently caught. For snow crab and Alaska pollock, a strong positive correlation was found (Figs S6.3 and S6.4 in S1 File), indicating that search volume is significantly higher in regions with greater catch volumes. For the remaining three species, no significant correlation was observed, suggesting that they are widely searched across Japan, regardless of regional catch volumes (Figs S6.5-S6.7 in S1 File).

### 3.4 Analysis of related search keywords

Across all seven fish species, search queries were primarily related to price information and cooking methods. Additionally, distinct cultural, regional, and seasonal factors were reflected in the search keywords for each species (S1-S7 Tables). “Setsubun” recurrently accompanied sardine queries (reflecting the hiiragi-iwashi custom); horse mackerel showed limited keyword diversity with modest regional-branding interest; squid and snow crab terms often overlapped with related species and processed products (and, for crab, strong e-commerce intent); Alaska pollock queries emphasized product names and regional name variants; and mackerel queries centered on processed or recipe-specific items (e.g., canned, miso-simmered). Notably, Pacific saury queries increasingly referenced catch declines and poor harvests, indicating heightened public concern over rapid stock depletion.

### 3.5 Path analysis

**Fig 2:**
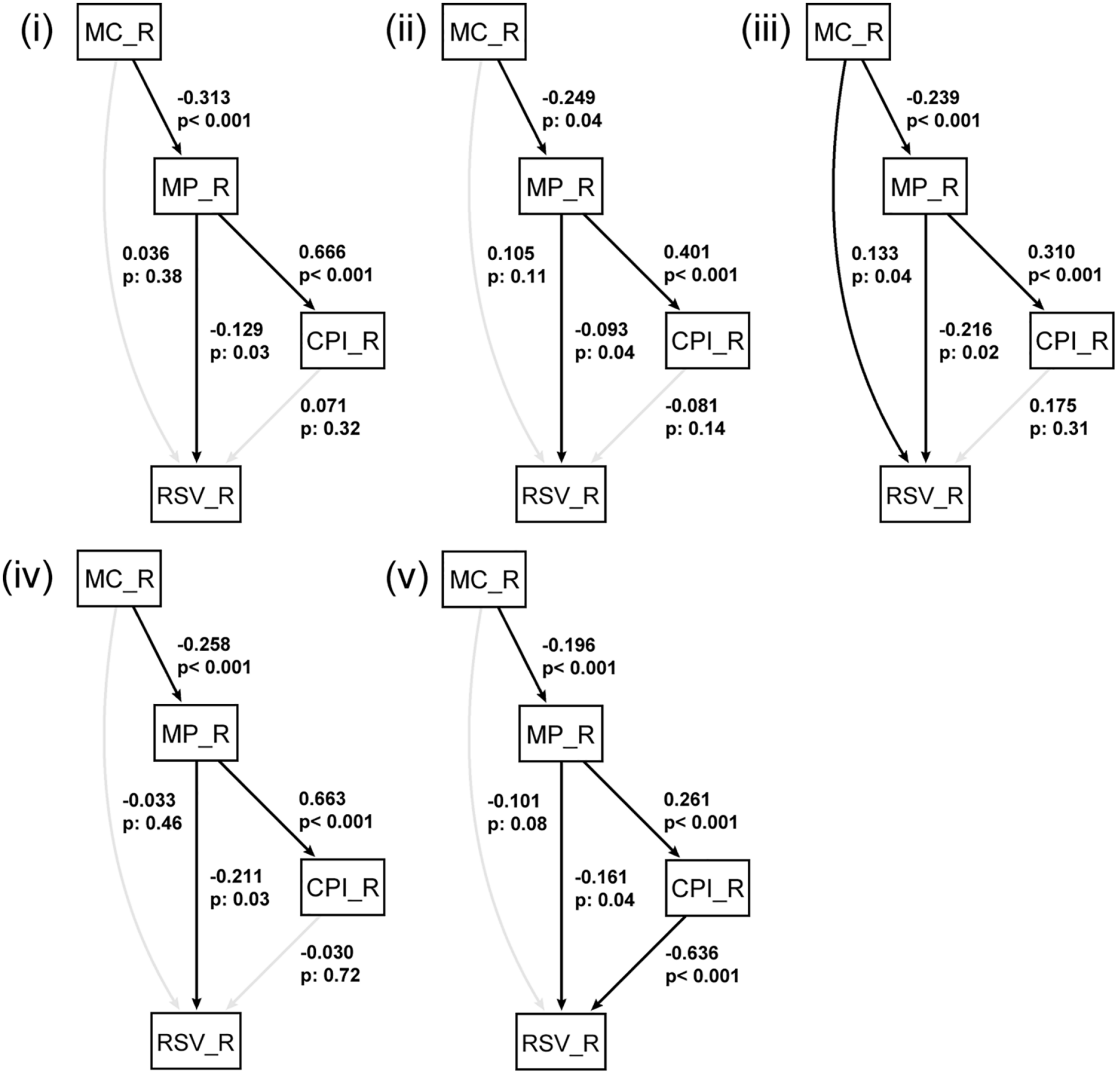
Results of path analysis. (i) Horse mackerel, (ii) Japanese sardine, (iii) Pacific saury, (iv) Mackerel, (v) Japanese flying squid. Statistically significant paths (p < 0.05) are indicated with higher opacity. Path analysis was applied to monthly data from 2010 to 2023 for all species.

Path analysis demonstrated a strong model fit for Japanese horse mackerel, Japanese sardine, Pacific saury, and mackerel species (Table 1). The estimated relationships are illustrated in Fig 2. The results indicated that market price at the central wholesale market primarily influenced search volume across these species. Additionally, Pacific saury was the only species where fishery catch volume had a significant, albeit small, impact on search volume. For Japanese flying squid, the model fit was lower compared to other species. However, both CPI and market price contributed to search volume, with CPI having a greater influence than market price, suggesting that consumer price fluctuations play a more significant role in search behavior for Japanese flying squid.

**Table 1:** Model fit indices for path analysis.

	DoF	χ² (p-value)	CFI	AGFI	NFI	TLI	RMSEA	AIC	BIC
Horse mackerel	2.00	0.41 (0.81)	1.00	0.99	1.00	1.03	< 0.01	16.00	40.99
Japanese sardine	2.00	2.16 (0.34)	0.98	0.89	0.97	0.99	0.02	15.97	40.97
Pacific saury	2.00	0.22 (0.90)	1.00	0.99	1.00	1.07	< 0.01	16.00	40.99
Mackerel	2.00	1.42 (0.49)	0.99	0.97	0.99	1.01	< 0.01	15.98	40.97
Japanese flying squid	2.00	6.15 (0.05)	0.93	0.77	0.93	0.83	0.11	15.93	40.92

This table reports DoF, χ² (p-value), CFI, AGFI, NFI, TLI, RMSEA, AIC, and BIC for each species. As general guides, CFI/TLI ≥ 0.90 and RMSEA ≤ 0.08 indicate acceptable fit; we emphasize patterns across indices rather than strict cutoffs.

(i) Japanese Horse Mackerel
Search volume (RSV_R) exhibited a significant negative association with market price (MP_R) (β = −0.129, p = 0.03), indicating that a decline in market price leads to an increase in search volume. In contrast, the effects of fishery catch volume (MC_R) and consumer price index (CPI_R) were not statistically significant (Fig 2. (i)).
(ii) Japanese Sardine
Search volume (RSV_R) was significantly influenced by market price (MP_R) (β = −0.093, p = 0.04), indicating that a decrease in market price is associated with an increase in search volume. Meanwhile, fishery catch volume (MC_R) and CPI (CPI_R) had no statistically significant effects (Fig 2. (ii)).
(iii) Pacific Saury
Search volume (RSV_R) exhibited a significant negative association with market price (MP_R) (β = −0.216, p = 0.02), confirming that a decline in market price is associated with an increase in search volume. Additionally, fishery catch volume (MC_R) had a significant positive effect on search volume (β = 0.133, p = 0.042), suggesting that an increase in catch volume leads to a rise in public interest. Conversely, CPI (CPI_R) did not have a significant effect (Fig 2. (iii)).
(iv) Mackerel Species
Search volume (RSV_R) was significantly influenced by market price (MP_R) (β = −0.211, p = 0.01), indicating that a decline in market price is associated with an increase in search volume. However, fishery catch volume (MC_R) and CPI (CPI_R) did not exhibit significant effects (Fig 2. (iv)).
(v) Japanese Flying Squid
Search volume (RSV_R) exhibited significant negative associations with both CPI (CPI_R: β = −0.636, p < 0.001) and market price (MP_R: β = −0.161, p = 0.04), suggesting that a decrease in price is associated with an increase in search volume. However, the effect of fishery catch volume (MC_R) was not statistically significant, indicating a limited contribution to search behavior (Fig 2. (v)). Additionally, the model fit indices were relatively low, suggesting room for improvement in the model’s explanatory power.

### 3.6 Integrative summary

Taken together, these results position Pacific saury as a distinctive outlier among the seven species. Like Japanese horse mackerel, saury exhibits seasonal synchrony between catch and search peaks, yet it diverges from the others along three dimensions. First, saury is price-positive at the monthly scale (higher search interest when CPI is higher), with CPI often remaining elevated around the September peak when catch, demand, and supply rise simultaneously. Second, in the path analysis, saury is the only species for which fishery catch volume has a significant direct (albeit small) positive effect on search interest, beyond the common negative price effect—indicating that availability itself amplifies public attention. Third, keyword patterns increasingly reference catch declines/poor harvests, signaling stock-status concerns that are less pronounced for other species. Consistent with this picture, prefecture-level coupling between catch and searches—strong for snow crab and Alaska pollock and weak for horse mackerel and sardine—was not evident for saury, suggesting nationally diffuse interest decoupled from local landings. Altogether, saury’s combination of seasonal synchrony, price-positive coupling, catch-driven attention, and stock salience marks it as qualitatively different from species whose search interest is primarily associated with lower prices or localized supply conditions.

## 4 Discussion

### 4.1 What drives public interest?

#### 4.1.1 Insights from time-series analysis and path analysis.

Seasonality emerged as a dominant, recurrent driver of search behavior, with pronounced annual peaks that align with cultural/culinary calendars (e.g., Japanese sardine with Hiiragi-Iwashi, Pacific saury in autumn, snow crab around year-end). These predictable cycles identify windows when outreach and public-facing campaigns are likely to be most effective.

Path analysis showed that price fluctuations affect search volume more than catch fluctuations. For Japanese horse mackerel, Japanese sardine, Pacific saury, and mackerel species, lower wholesale prices were significantly associated with higher search activity, whereas CPI effects were not significant. Wholesale prices primarily reflect domestic landing and distribution conditions, whereas the CPI aggregates retail-level prices across domestic and imported products; accordingly, this pattern accords with evidence that Japanese consumers prefer domestically sourced seafood [46].

A unique finding for Pacific saury was that search volume was influenced not only by market price but also by catch volume. This is likely due to the Pacific saury’s strong seasonality, which makes fluctuations in catch volume more newsworthy and easily captures consumer interest. In contrast, for other fish species, no significant relationship was observed between catch volume and search volume. This suggests that for these species, market price fluctuations drive consumer interest, while direct concern for catch conditions remains relatively low.

#### 4.1.2 Insights from the relationship between prefecture-level catch volumes and search volumes.

At the prefecture level, links between search and catch volumes differed by species, indicating that regional fishing and distribution shape consumer interest. For Japanese horse mackerel and Japanese sardine, correlations were weakly positive, with search hotspots in southern (horse mackerel) and northern (sardine) prefectures—consistent with their status as staples whose interest is higher where catches are frequent. For snow crab and Alaska pollock, correlations were strongly positive: searches clustered along the Sea of Japan coast (crab) and in northern prefectures (pollock), and time-series patterns plus co-search terms indicate occasional species confusion and that domestic catch signals can be obscured by external supply factors.

When disseminating information, and unless the objective is basic recognition, prioritizing high-catch/high-interest prefectures is likely the most efficient strategy under resource constraints. Species with pronounced regional attention can serve as regional flagships alongside a national flagship such as Pacific saury. Japan’s Pride Fish Project—an industry-led program that designates prefecture-specific emblematic species and promotes them through coordinated seasonal campaigns and information—provides a place-based template [21]: by elevating local species, it links cultural identity, local fisheries, and market communication. Leveraging such initiatives enables agencies and cooperatives to tailor messages to high-catch/high-attention prefectures and to deploy regional flagships as complements to Pacific saury.

#### 4.1.3 Insights from related search keywords.

Among the analyzed fish species, Pacific saury exhibited a unique trend, with an increasing number of searches related to catch volume. The results of the path analysis further confirmed that catch volume directly influences search volume for Pacific saury, making it the only species where consumer interest appears to be directly linked to fluctuations in catch volume. While the extent to which consumers are aware of the critical state of Pacific saury stocks remains debatable, these findings suggest that, given the low baseline interest of fishery stock conditions, Pacific saury may serve as an entry point for increasing public interest in sustainable fisheries management.

In contrast, for all other fish species, searches related to catch volume were minimal. Given that most of the species examined in this study face significant challenges in fisheries and resource management, it remains crucial to raise consumer awareness of these issues to ensure the long-term sustainability of fisheries.

### 4.2 Can pacific saury serve as a flagship species?

Based on the findings above, Pacific saury has emerged as a species that both reflects the recent deterioration of fishery resources and commands broad public attention, making it well suited as a flagship for outreach and management communication. Despite an overall downward trend, saury remains one of the most-searched species (second only to mackerel), and prefecture-level analyses show minimal regional disparity—indicating nationwide recognition rooted in Japan’s fish-eating culture and in its role as an indicator of the sector’s decline. Unlike most species, saury exhibits distinct supply–demand–price dynamics: whereas higher catches typically coincide with lower CPI and increased searches, saury shows its highest CPI and search volume in September, when both supply and demand peak, suggesting that domestically harvested autumn saury is ascribed special seasonal value rather than being treated merely as a low-cost staple. This interpretation aligns with evidence that food-related information seeking is stimulated not only by economic cues but also by health/safety, convenience, cultural/affective meanings, and ethical considerations [47]. Taken together, this combination of national salience, cultural meaning, and economic relevance provides practical leverage for using saury as a flagship species to focus communications, frame stock-status messaging, and time seasonal campaigns that promote sustainable fisheries.

The concept of flagship species has traditionally focused on charismatic megafauna such as tigers, elephants, and pandas [27,32]. More recently, flagship species have been increasingly recognized as strategic marketing tools aimed at influencing consumer behavior and fostering conservation efforts [32,47]. Building on this idea, the concept of “flagship events” extends the flagship-species approach by treating attention-grabbing natural or human-driven events as comparable levers; for example, recurrent seasonal phenomena can function as flagship events that draw audiences into biodiversity challenges and potentially mobilize support [48]. From this perspective, Pacific saury, as both a cultural icon and a species reflecting the challenges of fishery resource management, is well suited for media campaigns promoting sustainable fisheries. Species awareness-day type campaigns can measurably increase online information seeking and, when paired with explicit calls to action, can also strengthen engagement outcomes [49].

Considering these factors, Pacific saury has strong potential to serve as a flagship species for promoting sustainable fisheries in Japan. Its high societal recognition, cultural significance, economic value, and consumer interest in its declining catch volumes make it a compelling candidate for raising awareness of sustainable fisheries. However, sustaining long-term public interest and linking this awareness to policy initiatives require concrete strategic efforts. This is particularly relevant given persistent shortfalls in biodiversity-related media attention often without deliberate framing and amplification [50]. By leveraging the findings of this study and positioning Pacific saury as a flagship species for sustainable fisheries, broader societal awareness and policy-driven initiatives can be effectively advanced.

Unlike many terrestrial flagships that can prompt direct, hands-on stewardship, a pelagic and widely traded food fish is unlikely to mobilize on-site conservation activities. If adopted as a flagship, Pacific saury would instead function through communication, markets, and governance: concentrating seasonal messaging, guiding menus and retail cues toward responsible options, encouraging sustainable consumption choices, reinforcing the perceived value and legitimacy of sustainable fisheries, and enlarging public attention from saury to additional species of concern. Social media should be treated as a central dissemination channel alongside search and news media, given its documented influence on food choice, purchase intention, and consumption behavior [51]. In this sense, the primary contribution lies in shaping awareness, preferences, and support for management measures rather than eliciting direct intervention on the ground.

## 5 Conclusions

This study shows that public interest in major fishery species is shaped by multiple, measurable drivers rather than a single mechanism. First, seasonality is a dominant and predictable driver, creating recurrent windows when audiences are more receptive to information. Second, price dynamics generally exert stronger effects on interest than catch fluctuations. Third, species with regionally concentrated fisheries and food cultures show localized amplification of interest (e.g., snow crab along the Sea of Japan coast and Alaska pollock in northern prefectures). Methodologically, combining Google Trends with routine statistics provides a transferable, lightweight way to integrate a societal-interest dimension into fisheries management and communication beyond Japan.

### 5.1 Policy and management implications

#### 5.1.1 Species-tailored communication using predictable attention.

National and prefectural fisheries authorities and co-management bodies should time public communication to recurrent seasonal peaks in public interest identified from multi-year search patterns. Scheduling advisories, outreach, and educational materials to these naturally receptive windows can maximize effects without additional cost. These recurrent peaks can be treated as predictable flagship-event windows for outreach planning.

#### 5.1.2 Strategic selection of intervention regions.

Alignment between local catches and local attention can be used to prioritize high-catch/high-attention prefectures when resources are limited. Place-based initiatives such as the Pride Fish Project are expected to foster sustained public interest in sustainable fisheries in specific regions, partly through associated local economic effects.

#### 5.1.3 Flagship-led uplift beyond a single species.

Use Pacific saury as a national flagship to raise attention to sustainable fisheries more broadly, not only to saury itself. The aim is to foster sustainable consumption choices (e.g., certified or responsibly sourced options), strengthen public appreciation of ongoing initiatives (such as the Pride Fish Project and community-based efforts), and attract evaluation and investment toward programs that demonstrate results. To translate attention into action, campaigns should be deliberately framed and disseminated across news and social media channels.

#### 5.1.4 From long-term monitoring to intervention evaluation.

While this study used search data to characterize long-term dynamics, the same indicators can support prospective assessment of specific interventions. Pre-specify evaluation metrics—for example, shifts in search attention during campaign windows, changes in sales composition toward responsibly sourced products, and brief awareness/understanding scores—and compare pre/post or treated vs. comparison areas.

### 5.2 Limitations and future work

Despite these limitations, the approach remains low-cost, scalable, and readily transferable. Properly timed to predictable peaks and adapted to local contexts, this methodology can support evidence-based outreach and policy design. Ultimately, integrating societal data into resource management is not just a technical exercise but a necessary step toward fostering a more engaged and informed public, capable of stewarding our shared marine heritage into the future.

Furthermore, a specific limitation of our study is that the path analysis model for Japanese flying squid did not achieve a good fit, unlike the models for other species. This suggests that the simple causal structure we hypothesized may not fully capture the complex drivers of public interest for this particular species. The demand for Japanese flying squid is heavily tied to the processed food industry, for example through dried-squid products, and its price and availability may be influenced by factors beyond domestic catch and consumer prices, such as import volumes and processing-industry trends. Future research—both for Japanese flying squid and more generally across species—should explore alternative model specifications and incorporate additional explanatory variables, including media-coverage intensity; social-media activity and engagement metrics; campaign and event indicators; market-side factors such as import volumes and processing-industry conditions; and, when relevant, indicators of seafood safety and environmental risks such as pollution incidents, contamination advisories, or food-safety concerns. Qualitative approaches, including semi-structured interviews with consumers, retailers and market actors, and fishery stakeholders, can be used to strengthen and contextualize the quantitative findings by clarifying how and why particular species attract public attention, especially in terms of perceived cultural and culinary value, consumption contexts, and their potential positioning as flagship species for sustainability communication.

## Supporting information

S1 File Supplementary figures and tables. Contains additional figures referenced in the main text (e.g., Figs S1.1), including correlation and residual analyses.
(DOCX)

S1 Table Google Trends keywords for Japanese horse mackerel (*Trachurus japonicus*). Japanese search keywords (original terms) for Japanese horse mackerel with English translations and annual Google Trends relative search volumes (2010–2023, Japan).
(CSV)

S2 Table Google Trends keywords for Japanese sardine (*Sardinops melanostictus*). Japanese search keywords (original terms) for Japanese sardine with English translations and annual Google Trends relative search volumes (2010–2023, Japan).
(CSV)

S3 Table Google Trends keywords for Pacific saury (*Cololabis saira*). Japanese search keywords (original terms) for Pacific saury with English translations and annual Google Trends relative search volumes (2010–2023, Japan).
(CSV)

S4 Table Google Trends keywords for Japanese flying squid (*Todarodes pacificus*). Japanese search keywords (original terms) for Japanese flying squid with English translations and annual Google Trends relative search volumes (2010–2023, Japan).
(CSV)

S5 Table Google Trends keywords for snow crab (*Chionoecetes opilio*). Japanese search keywords (original terms) for snow crab with English translations and annual Google Trends relative search volumes (2010–2023, Japan).
(CSV)

S6 Table Google Trends keywords for Alaska pollock (*Gadus chalcogrammus*). Japanese search keywords (original terms) for Alaska pollock with English translations and annual Google Trends relative search volumes (2010–2023, Japan).
(CSV)

S7 Table Google Trends keywords for mackerel species (*Scomber japonicus*; *Scomber australasicus*). Japanese search keywords (original terms) for mackerel species with English translations and annual Google Trends relative search volumes (2010–2023, Japan). Chub mackerel: *Scomber japonicus*; spotted mackerel: *Scomber australasicus*.
(CSV)
