# Supplementary material for: Analyzing and modeling public interest in fishery resources: Proposing flagship species for promoting sustainable fisheries in Japan
Source: PLoS One. 2026 Mar 9;21(3):e0342833. doi: 10.1371/journal.pone.0342833 (PMC12970879; doi:10.1371/journal.pone.0342833)
Supplement: S1 File — Contains additional figures referenced in the main text (e.g., Figs S1.1), including correlation and residual analyses. (DOCX) [file pone.0342833.s001.docx]

**Supplementary Materials**

Figure S1. STL Decomposition Results. Each panel includes the original data with its trend component (upper panel) and the seasonal (light blue line) and residual components (red bars) (lower panel). Results by fish species are shown below. (a) represents the decomposition results of monthly catch volumes (metric tons) from January 2010 to December 2023.　(b) represents the decomposition results of monthly search volumes (scaled to a maximum of 100) over the same period. Results by species are shown below. All figures are in the same format.


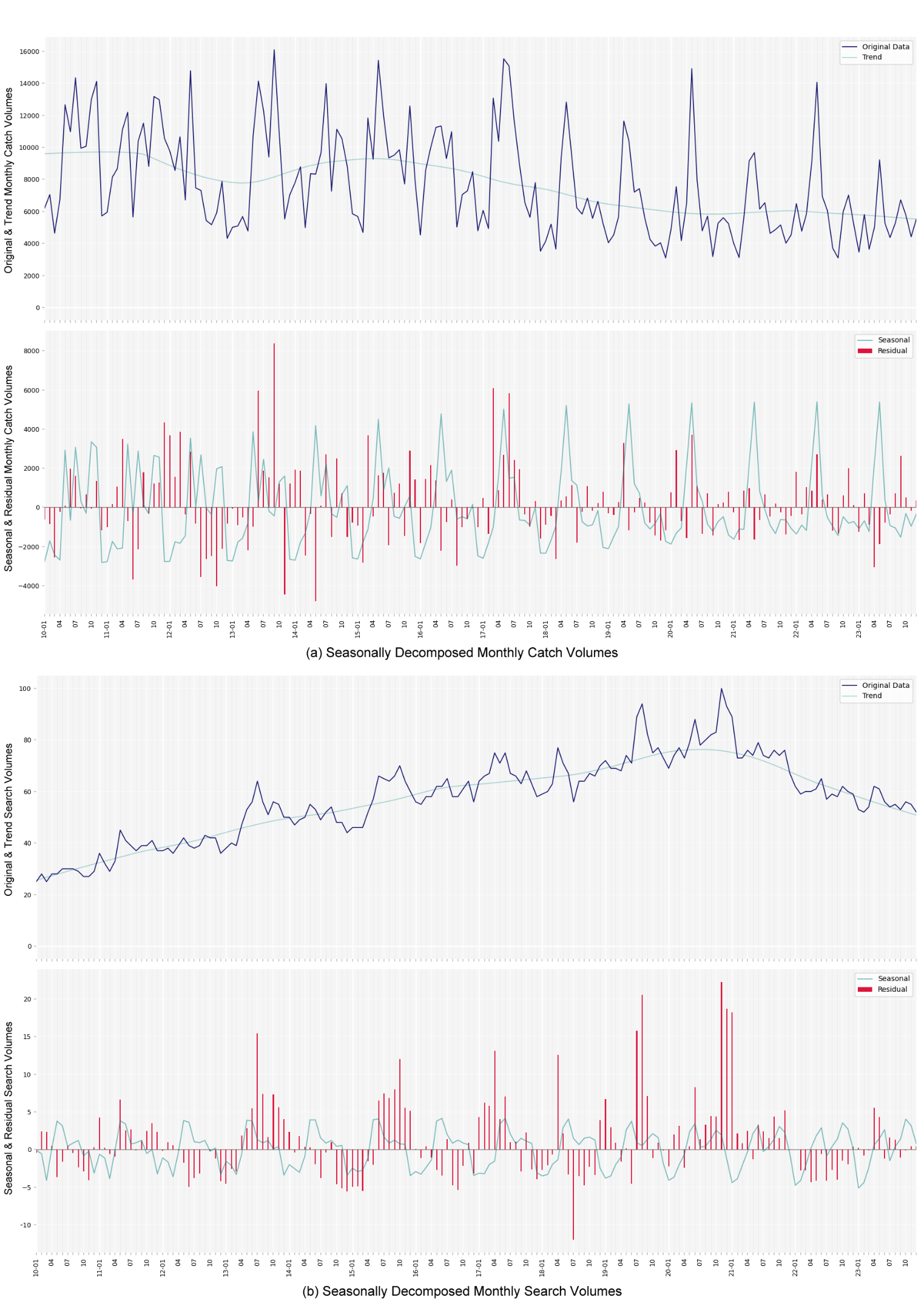


Figure S1.1. STL Decomposition Results for Horse mackerel (*Trachurus japonicus*).


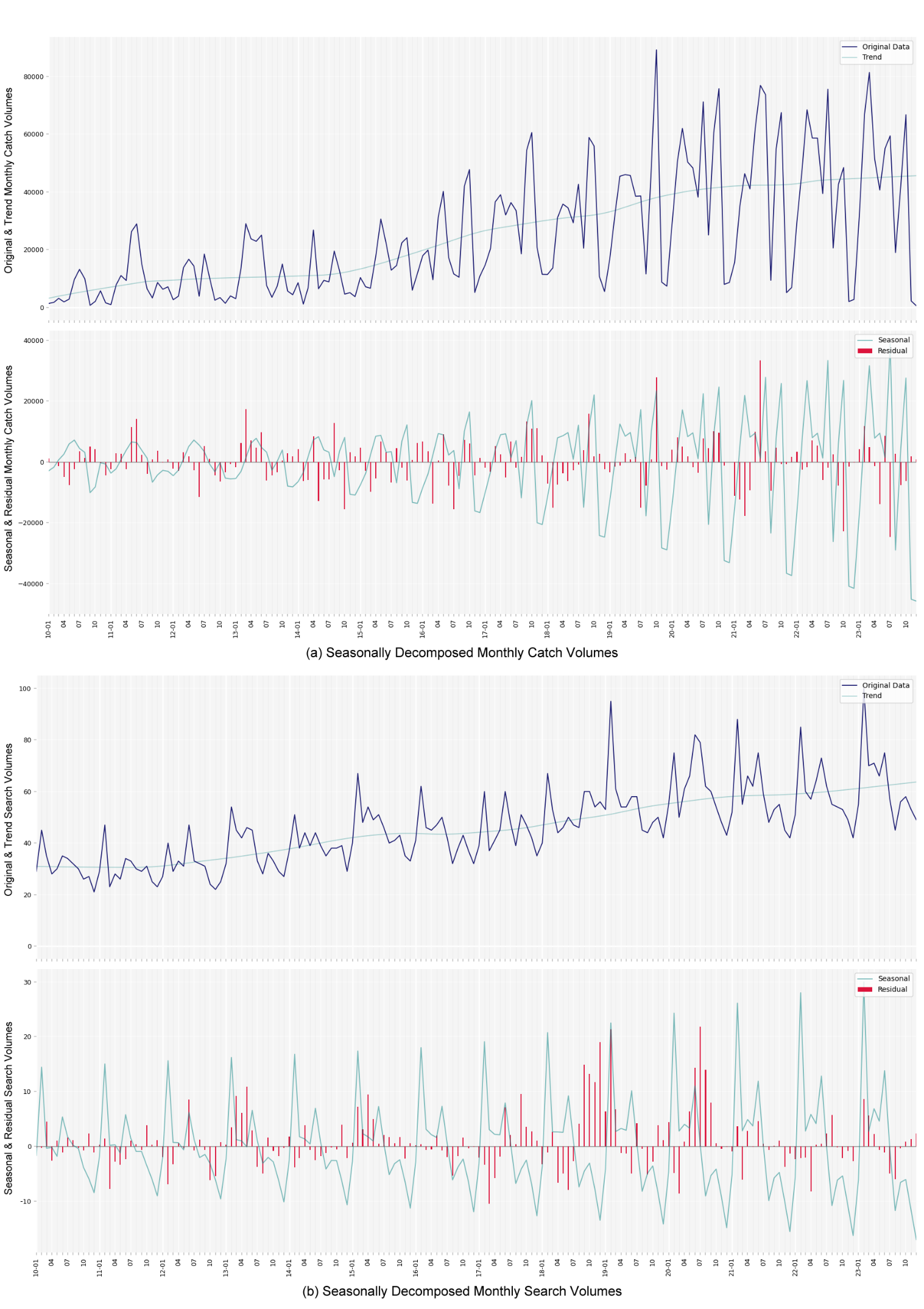


Figure S1.2. STL Decomposition Results for Japanese sardine (*Sardinops meknostictus*).


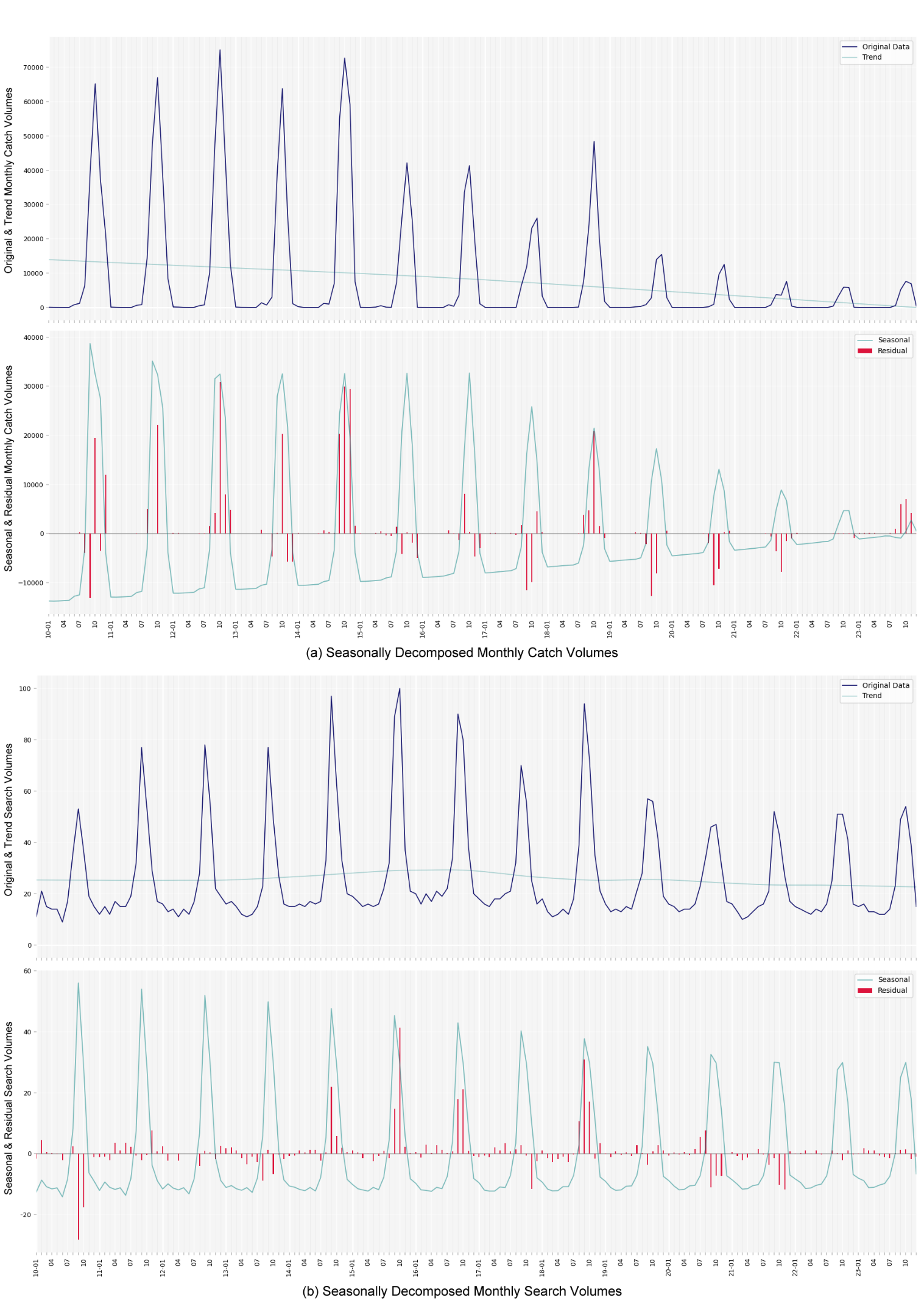


Figure S1.3. STL Decomposition Results for Pacific saury (*Cololabis Saira*).


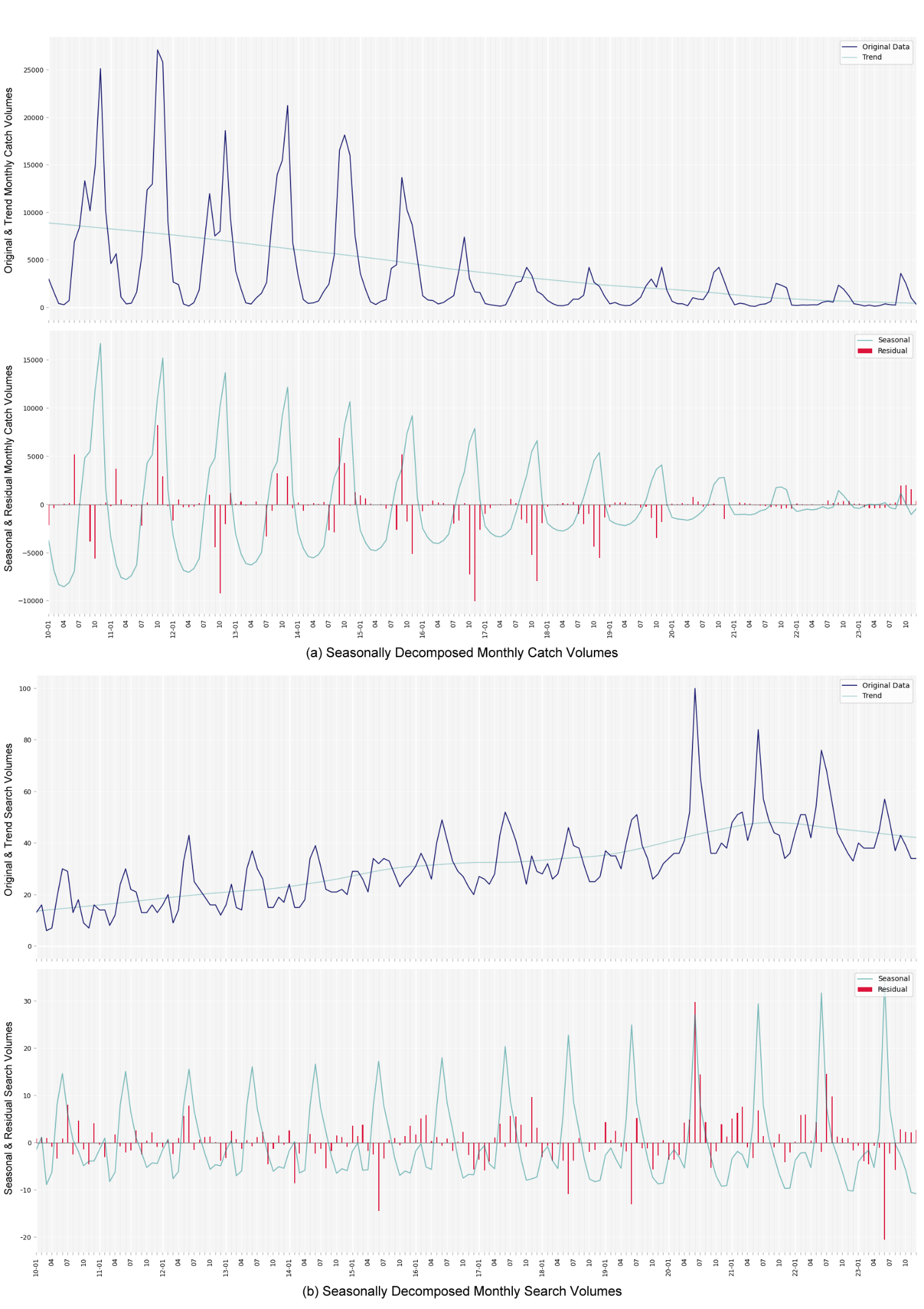


Figure S1.4. STL Decomposition Results for Pacific flying squid (*Todarodes pacificus*).


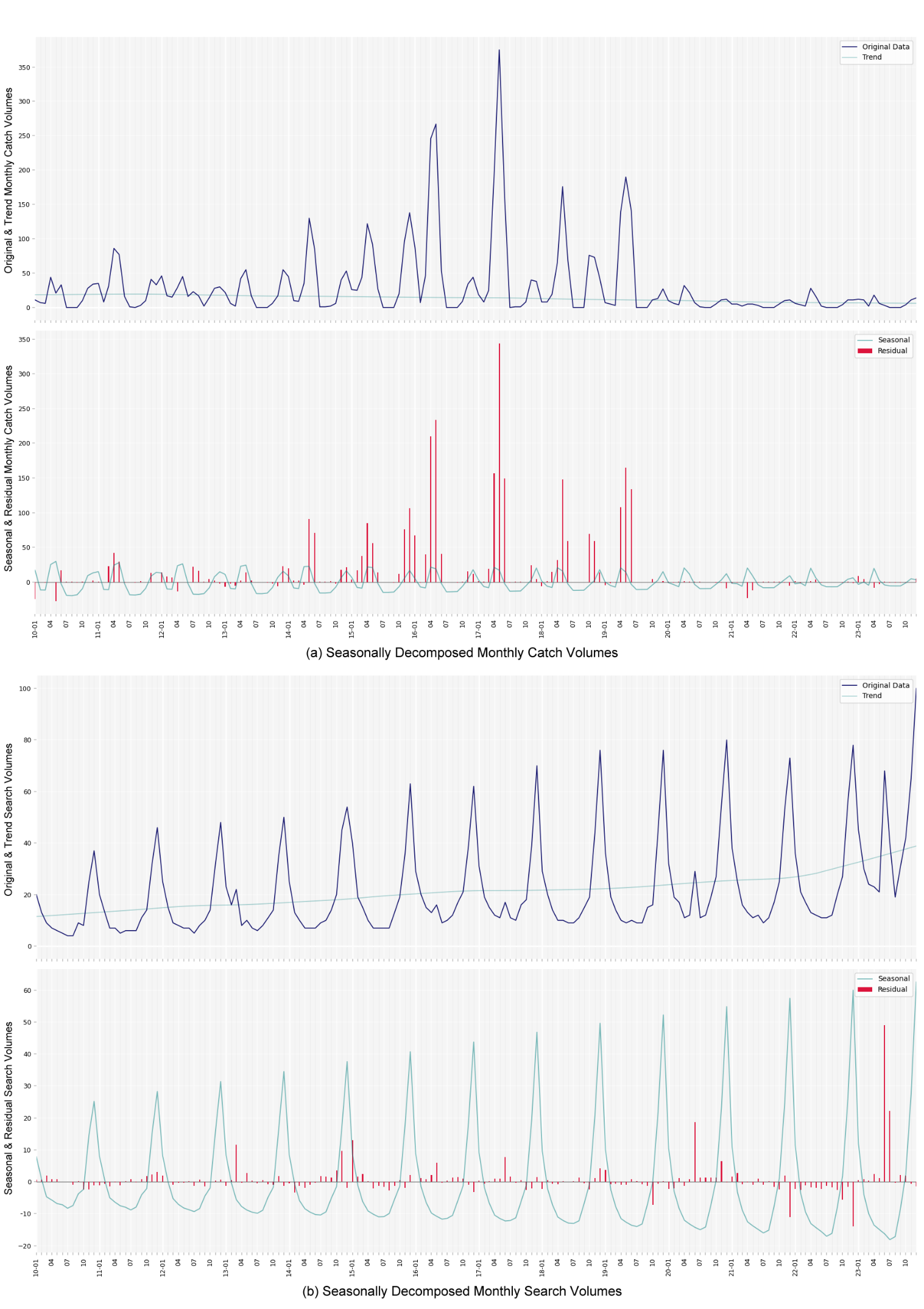


Figure S1.5. STL Decomposition Results for Snow crab (*Chionoecetes opilio*).


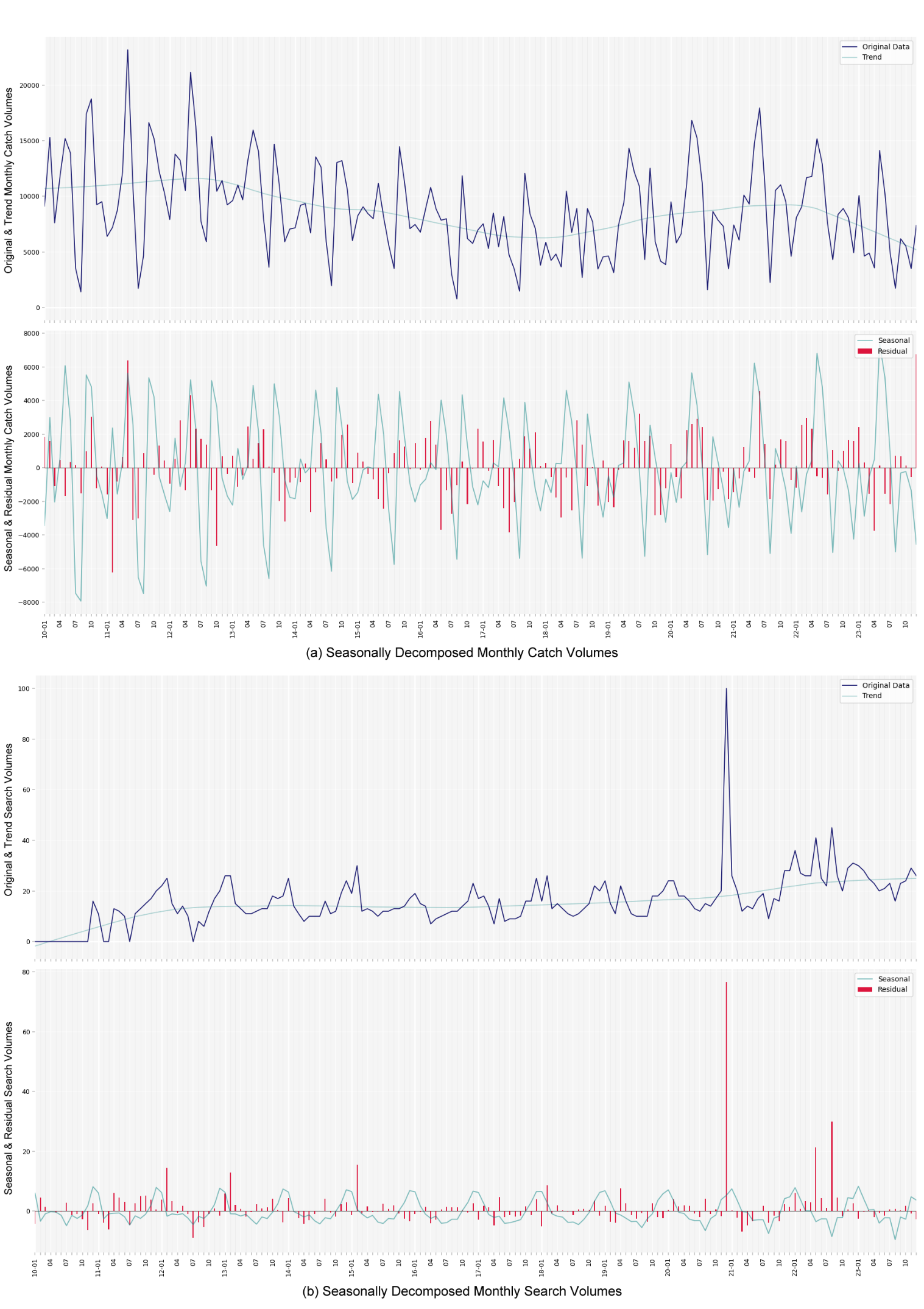


Figure S1.6. STL Decomposition Results for Alaska pollack (*Gadus chalcogrammus*).


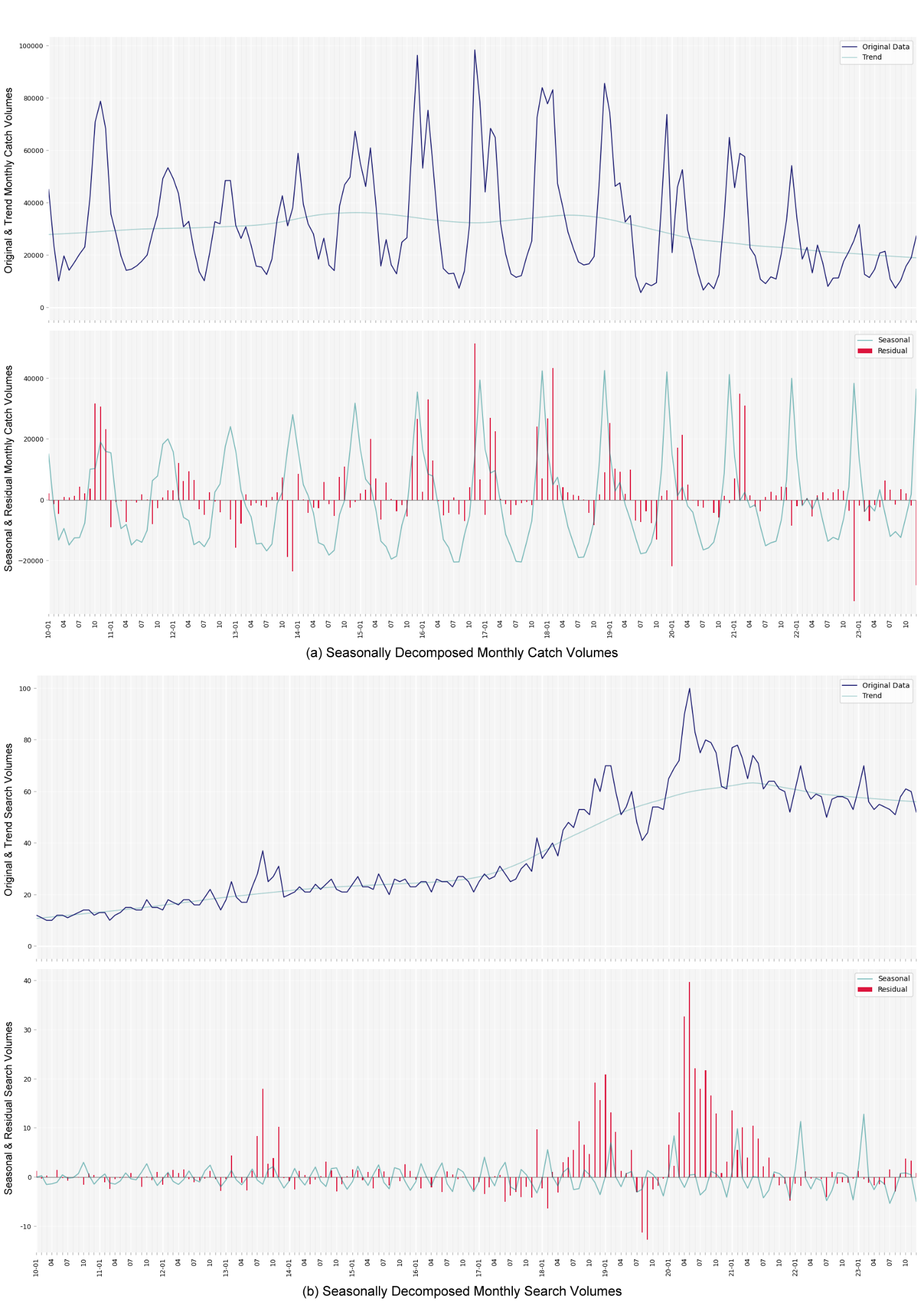


Figure S1.7. STL Decomposition Results for Mackerel (S*comber japonicus*, *Scomber australasicus*).

Figure S2. Relationship between de-trended and residual components of normalized monthly search volumes and monthly catch volumes. (a) shows the relationship using de-trended components, and (b) shows the relationship using residual components. The Spearman’s rank correlation coefficient (ρ) and its significance (P-value) are provided for each species. Darker points represent more recent data points. Results by species are shown below. All figures are in the same format.

*
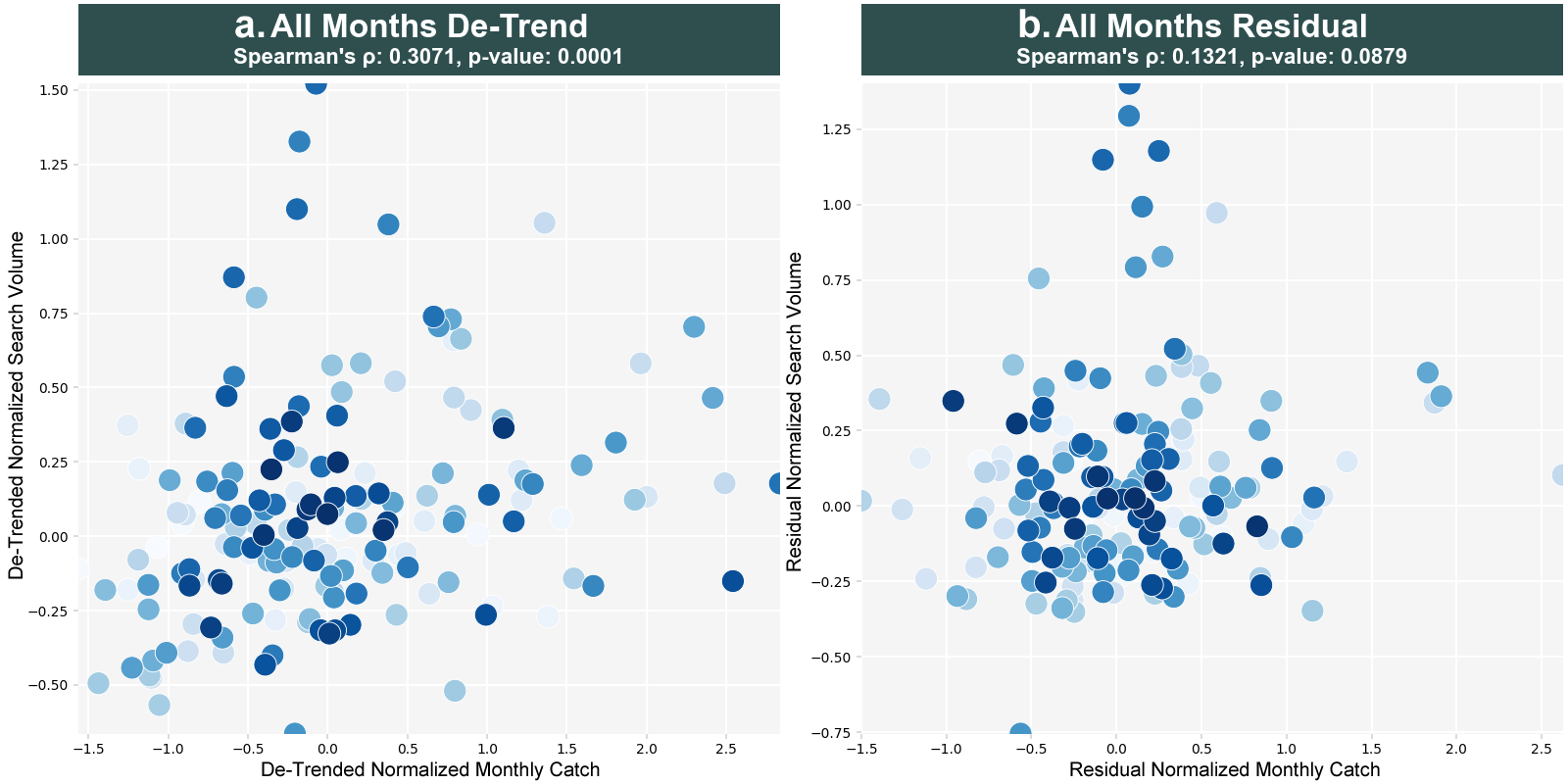
*

Figure S2.1. Relationship between de-trended and residual components of normalized monthly search volumes and monthly catch volumes for Horse mackerel (Trachurus japonicus).

*
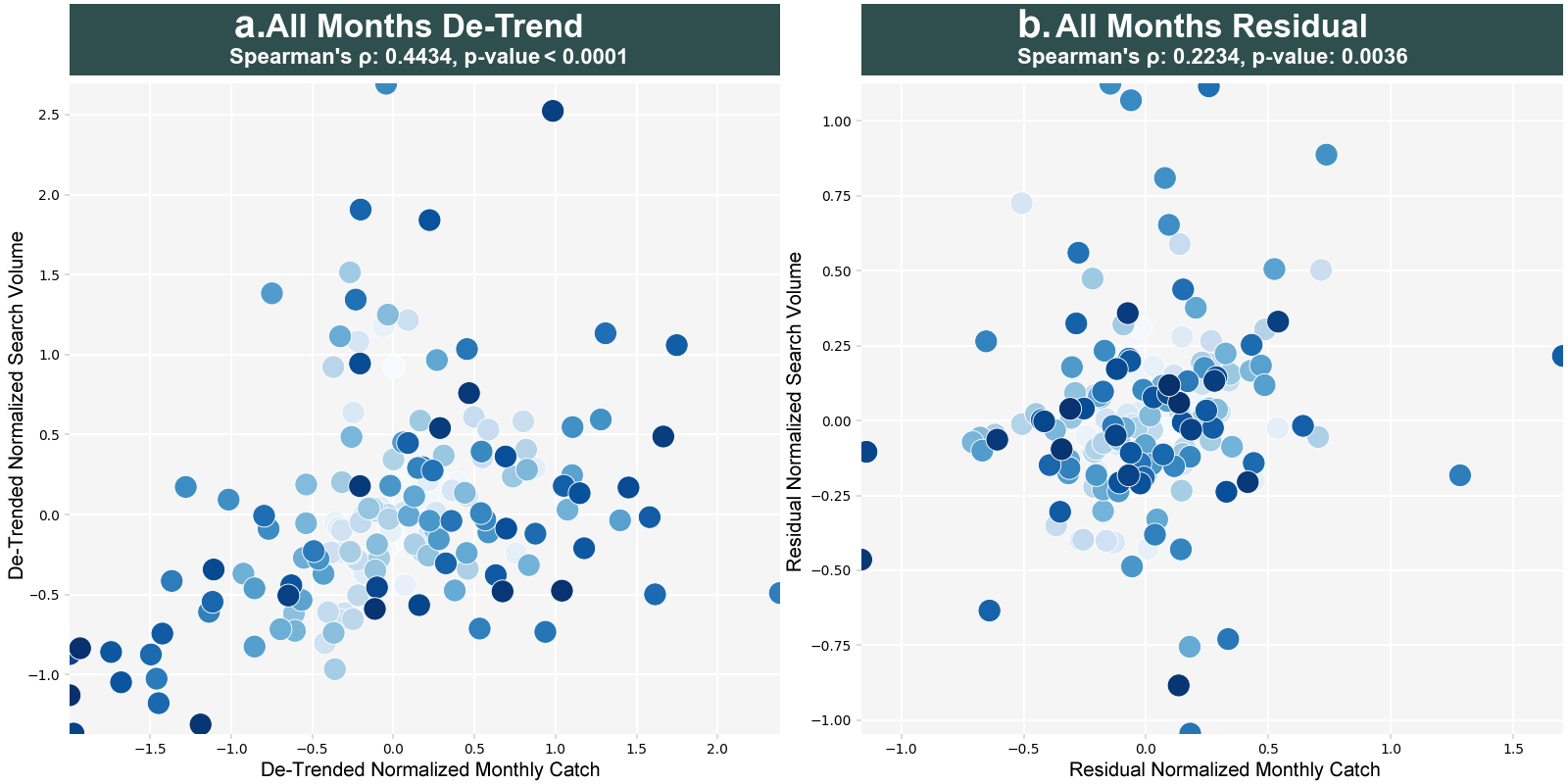
*

Figure S2.2. Relationship between de-trended and residual components of normalized monthly search volumes and monthly catch volumes for Japanese sardine (Sardinops meknostictus).

*
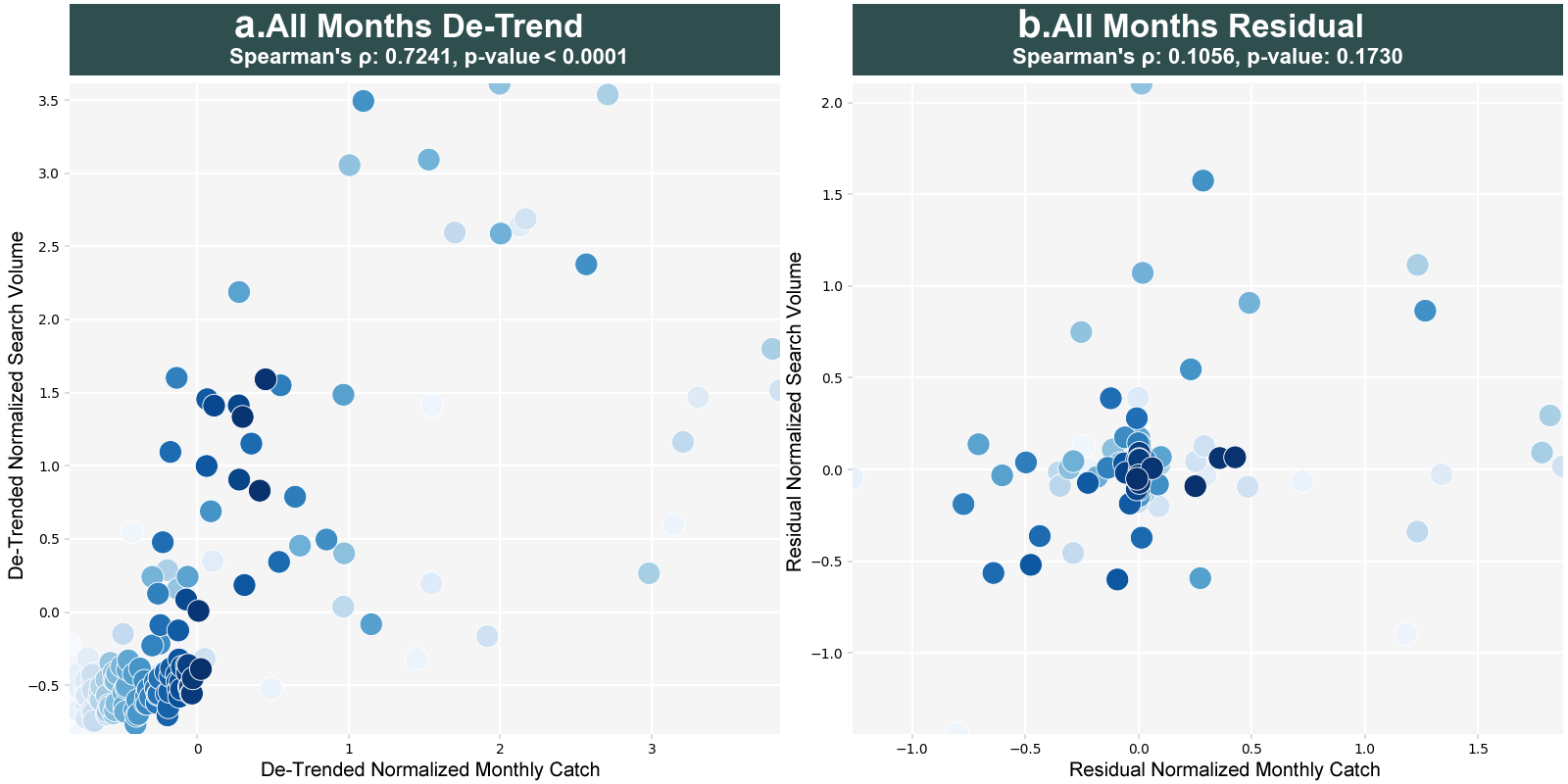
*

Figure S2.3. Relationship between de-trended and residual components of normalized monthly search volumes and monthly catch volumes for Pacific saury (Cololabis Saira).

*
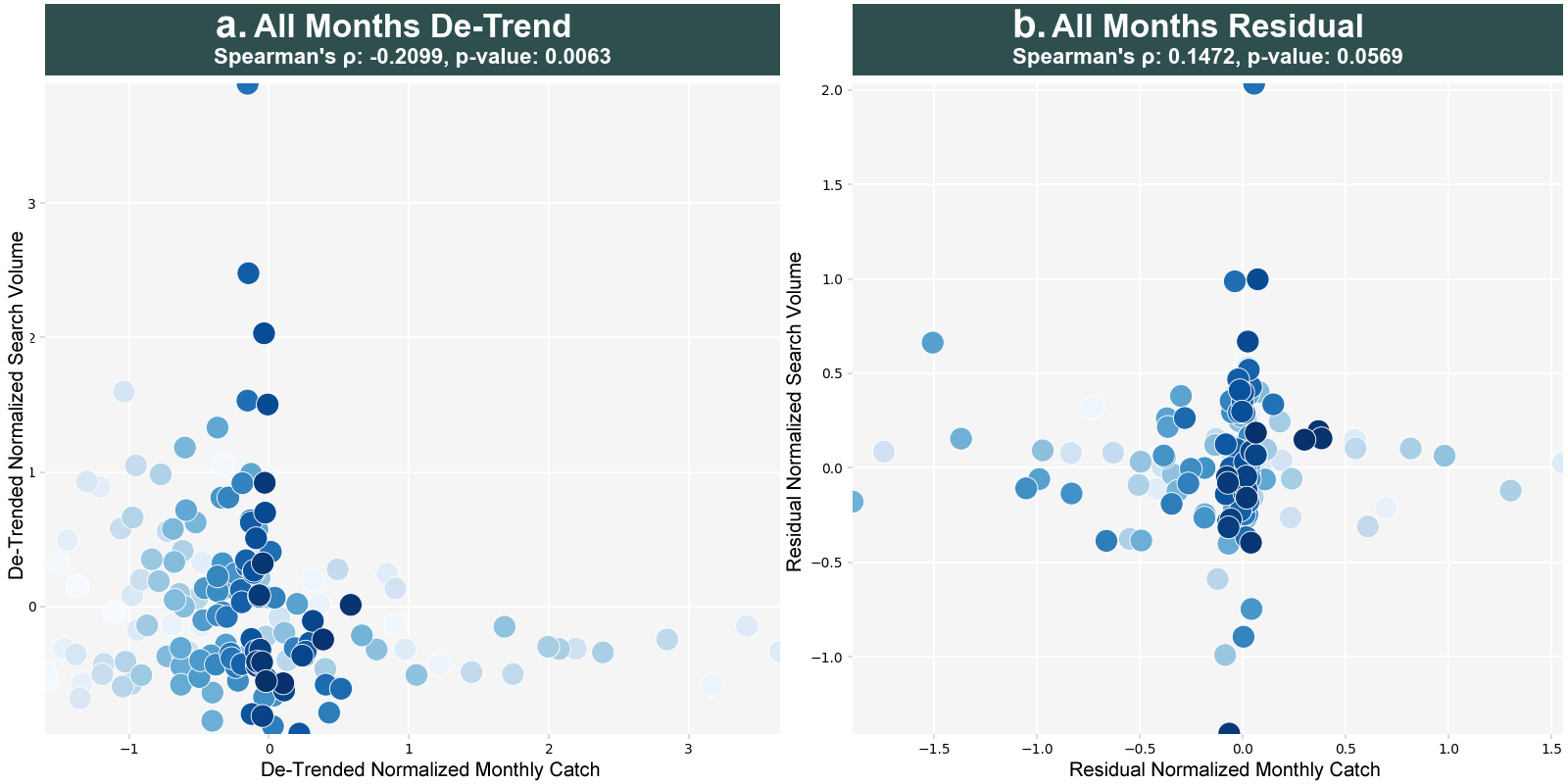
*

Figure S2.4. Relationship between de-trended and residual components of normalized monthly search volumes and monthly catch volumes for Pacific flying squid (Todarodes pacificus).

*
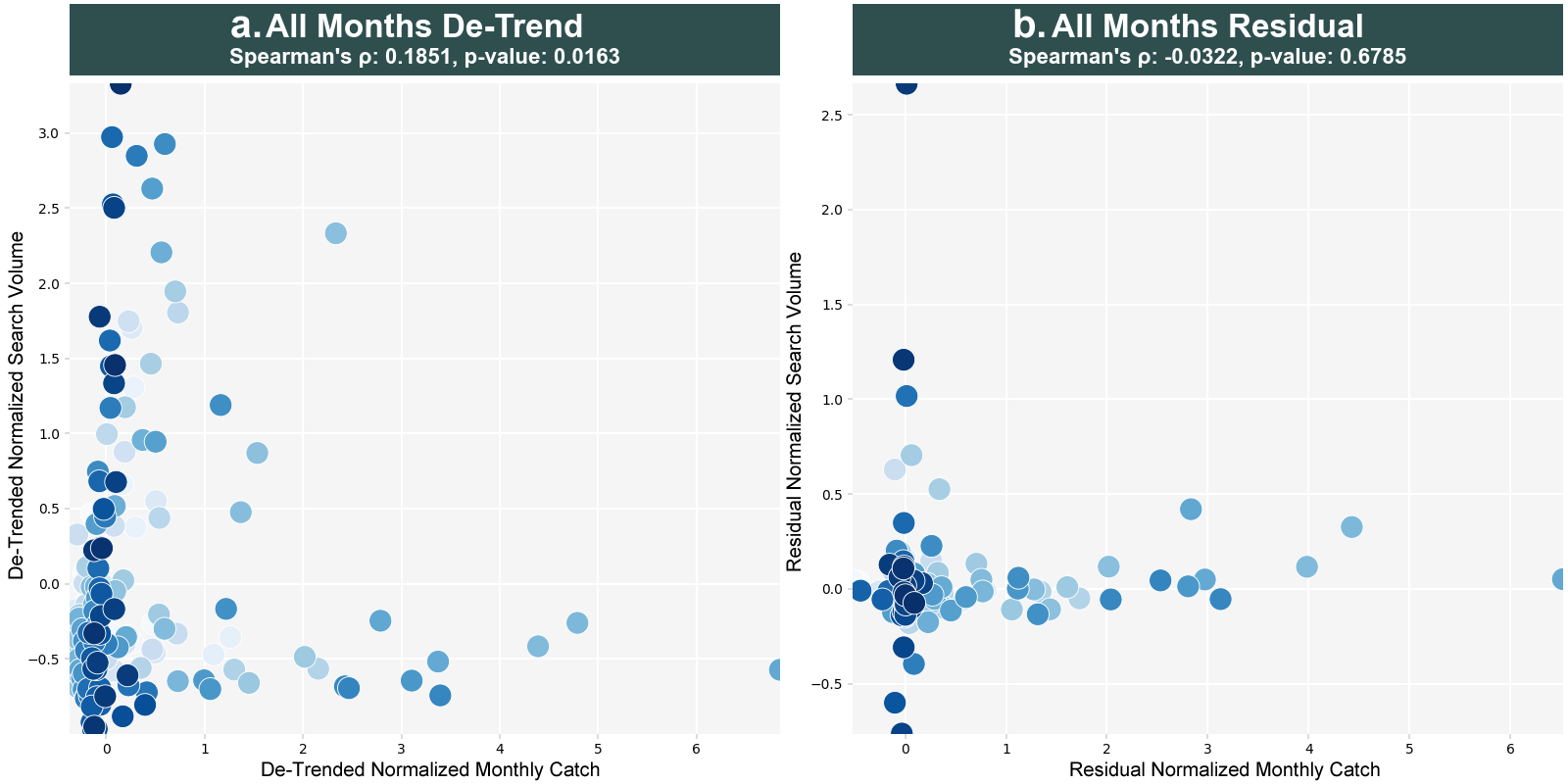
*

Figure S2.5. Relationship between de-trended and residual components of normalized monthly search volumes and monthly catch volumes for Snow crab (Chionoecetes opilio).

*
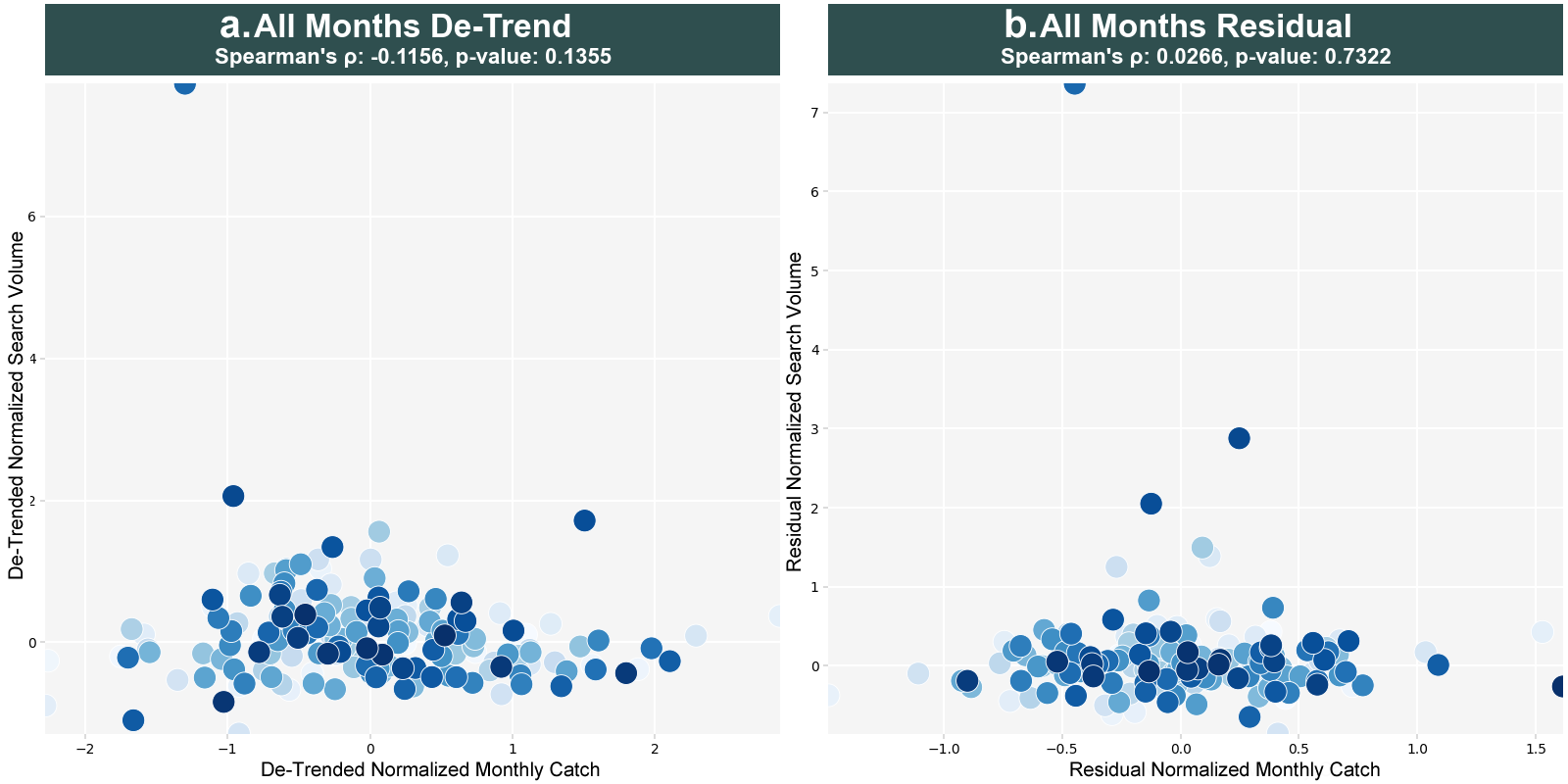
*

Figure S2.6. Relationship between de-trended and residual components of normalized monthly search volumes and monthly catch volumes for Alaska pollack (Gadus chal-cogrammus).

*
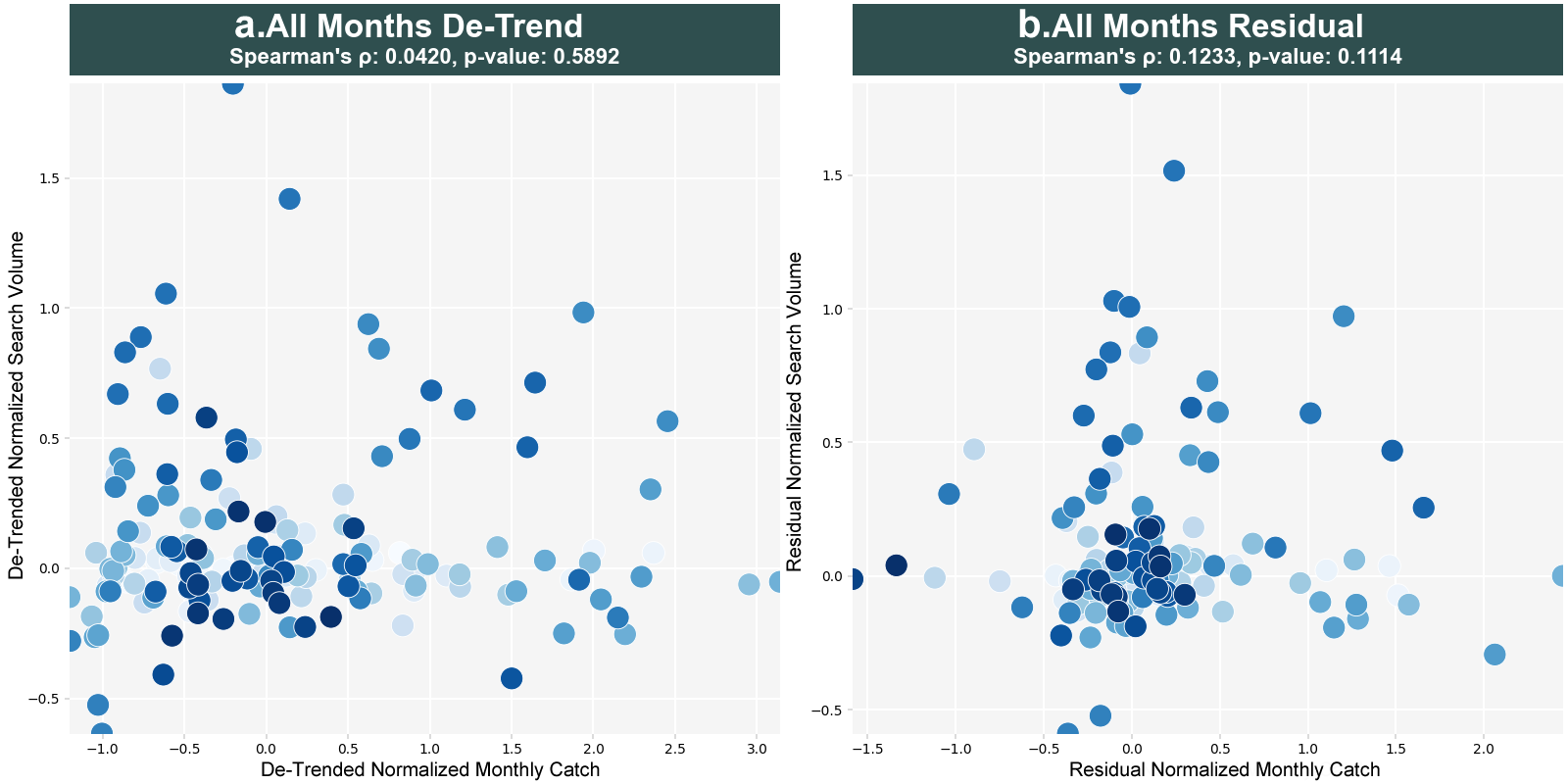
*

Figure S2.7. Relationship between de-trended and residual components of normalized monthly search volumes and monthly catch volumes for Mackerel (Scomber japonicus, Scomber australasicus).

*
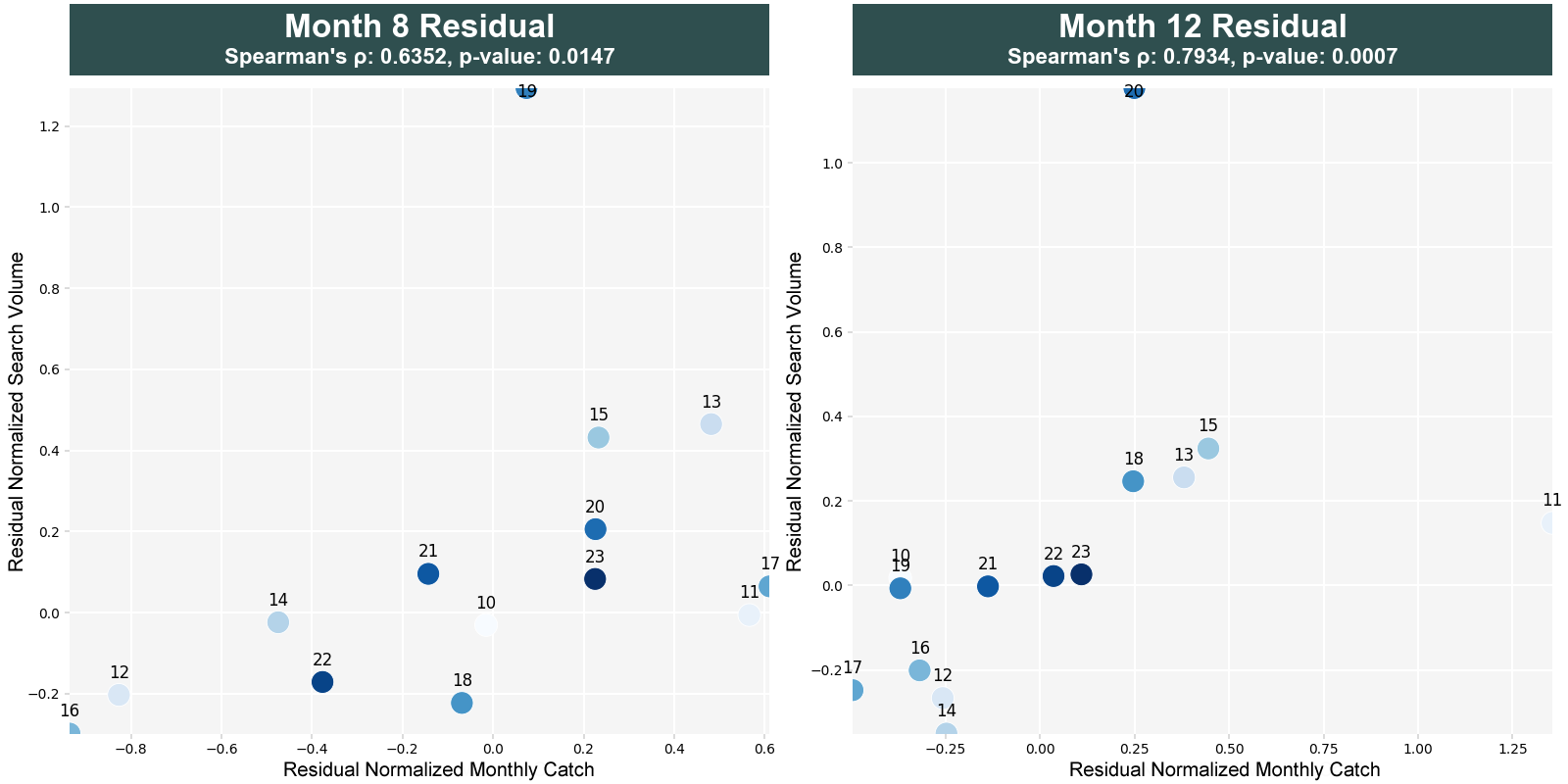
*

Figure S3.1. Relationship between residual components of normalized monthly search volumes and monthly catch volumes in August and December for Horse mackerel (Tra-churus japonicus).


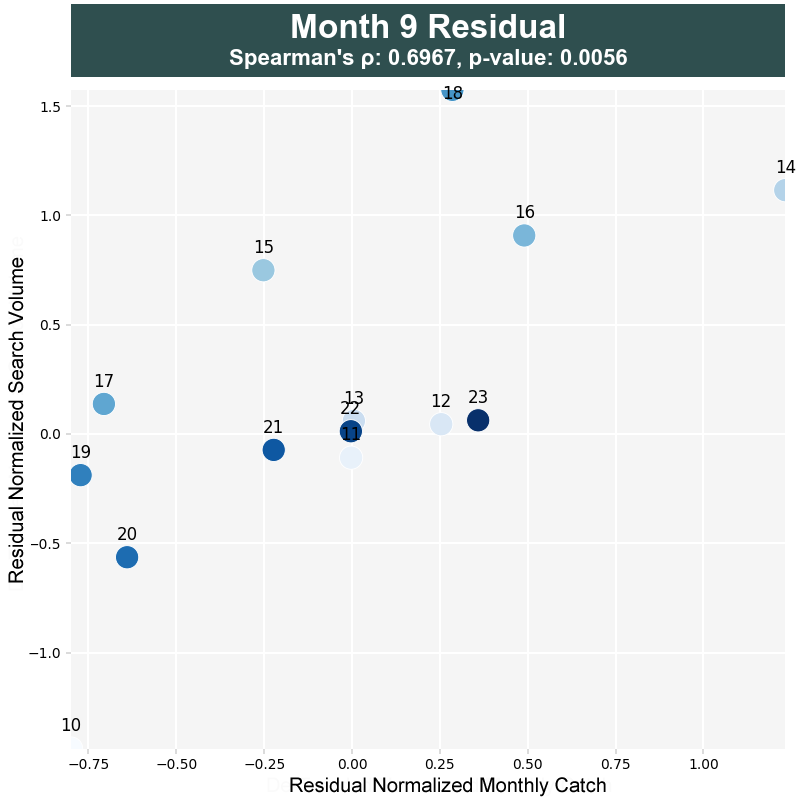


Figure S3.2. Relationship between residual components of normalized monthly search volumes and monthly catch volumes in September for Pacific saury (Cololabis Saira).

*
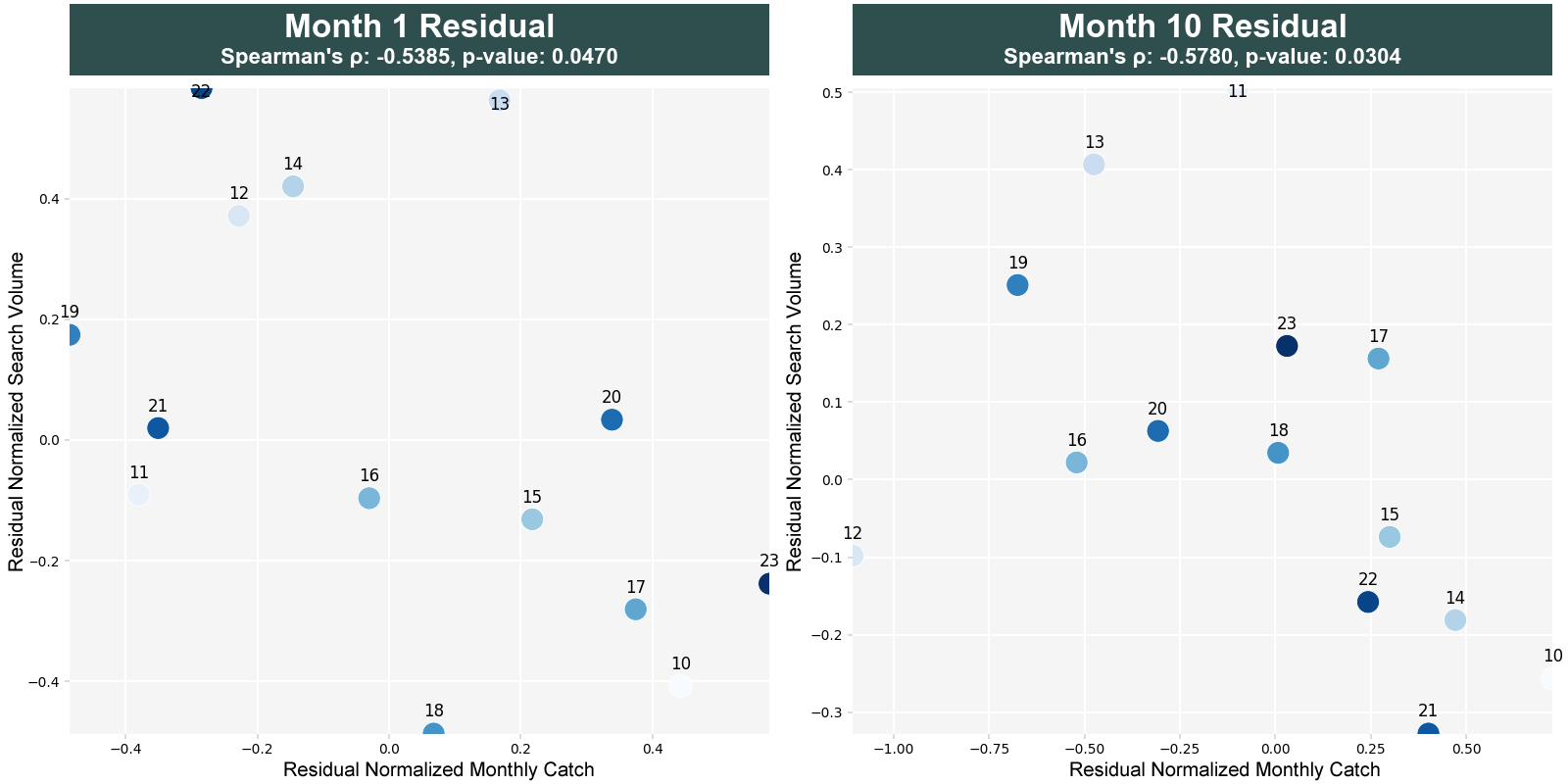
*

Figure S3.3. Relationship between residual components of normalized monthly search volumes and monthly catch volumes in January and October for Alaska pollack (Gadus chalcogrammus).

Fig. S4. Relationship between de-trended and residual components of normalized monthly search volumes and monthly CPI. (a) shows the relationship using de-trended components, and (b) shows the relationship using residual components. The Spearman’s rank correlation coefficient (ρ) and its significance (P-value) are provided for each species. Darker points represent more recent data points. Results by species are shown below. All figures are in the same format.

*
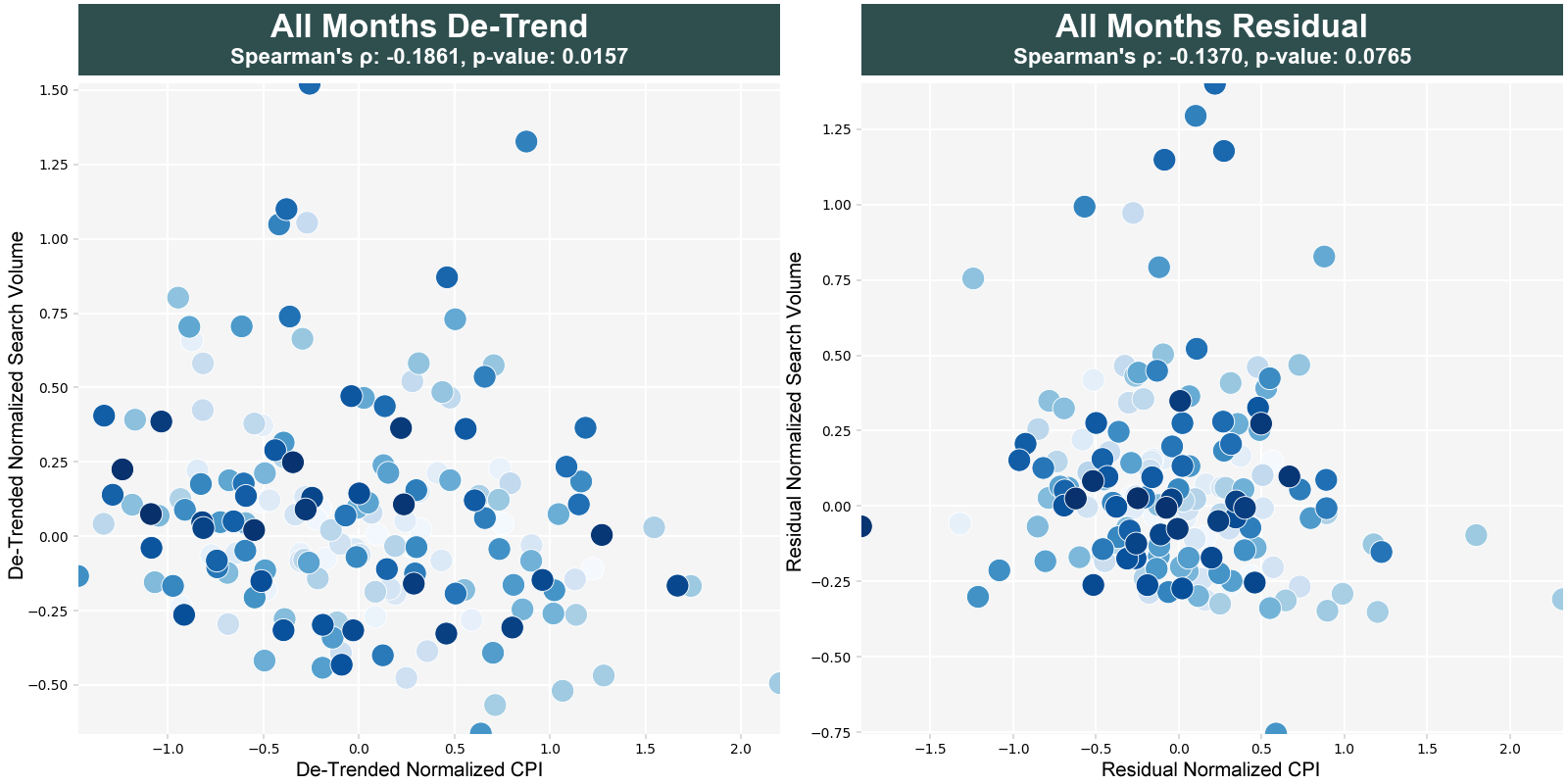
*

Figure S4.1. Relationship between de-trended and residual components of normalized monthly search volumes and monthly CPI for Horse mackerel (Trachurus japonicus).

*
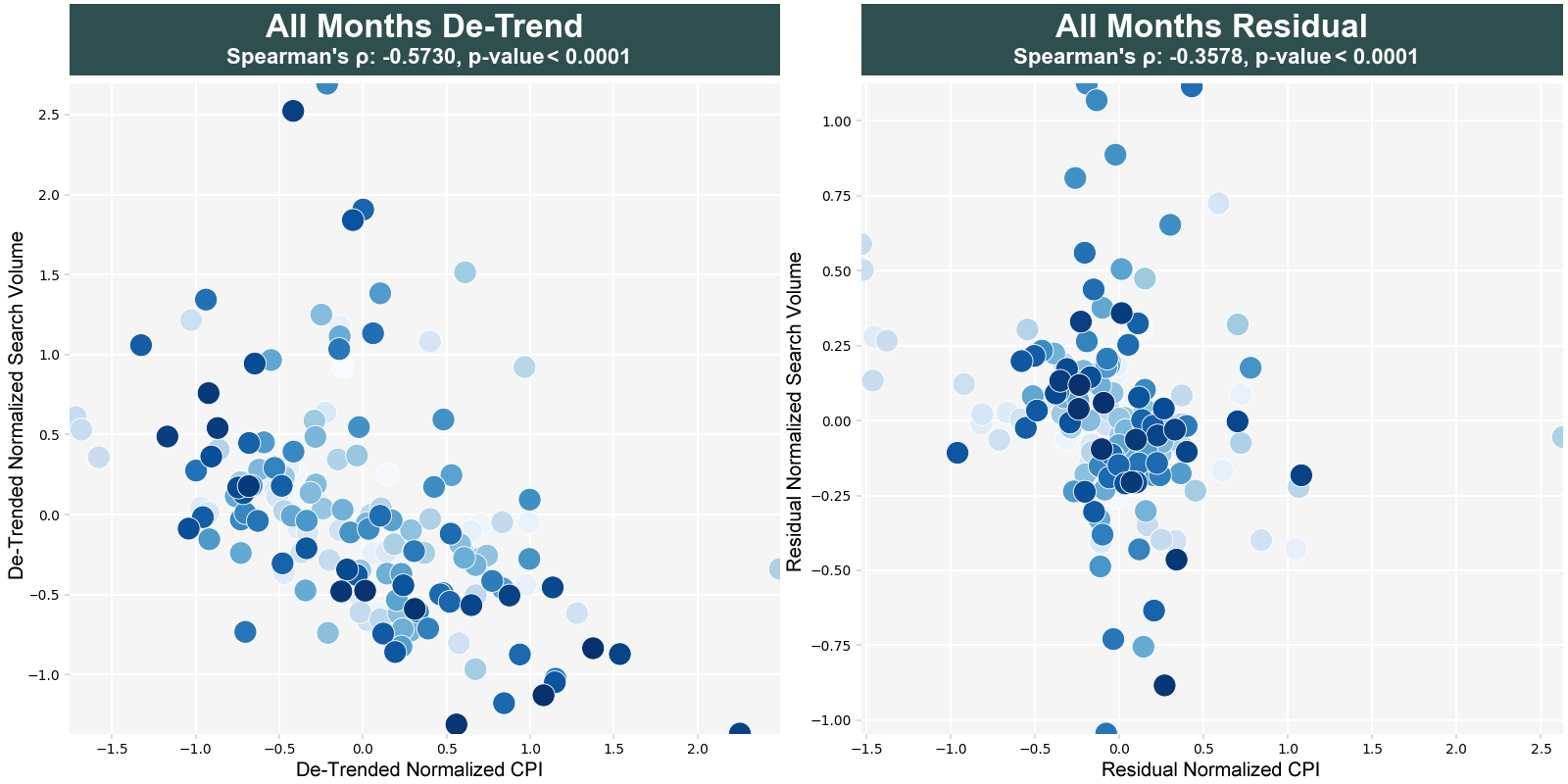
*

Figure S4.2. Relationship between de-trended and residual components of normalized monthly search volumes and monthly CPI for Japanese sardine (Sardinops meknostictus).

*
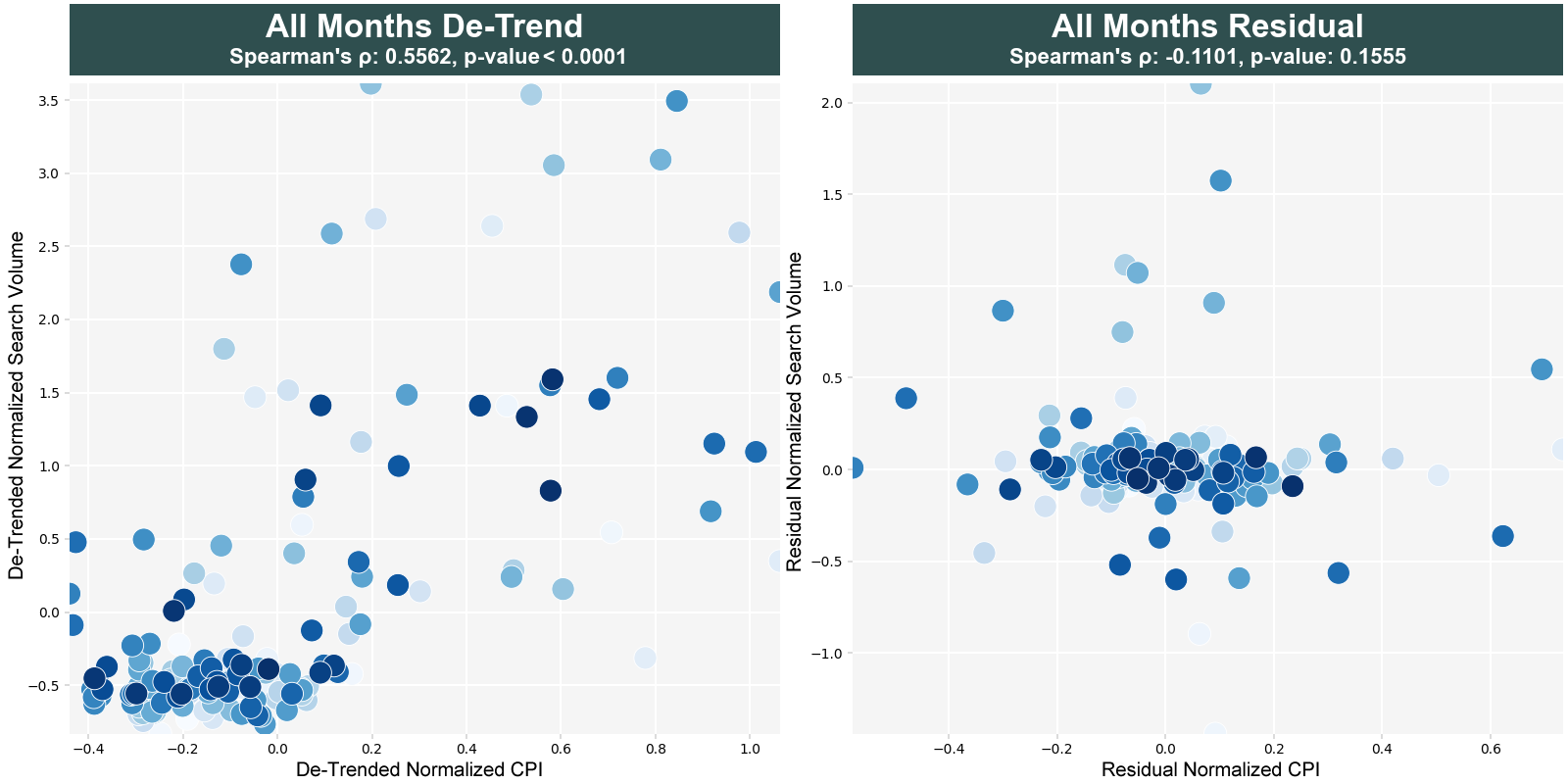
*

Figure S4.3. Relationship between de-trended and residual components of normalized monthly search volumes and monthly CPI for Pacific saury (Cololabis Saira).

*
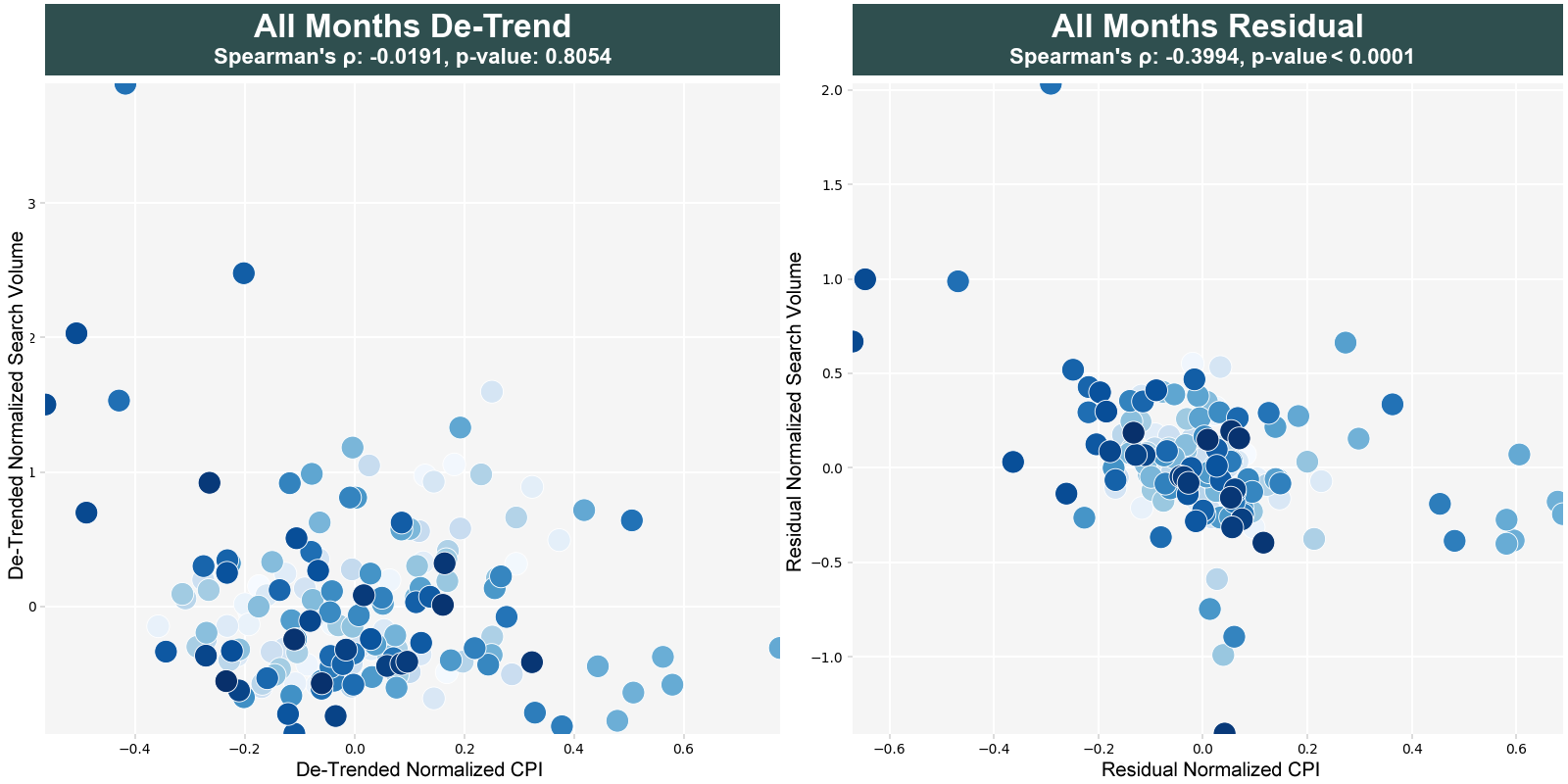
*

Figure S4.4. Relationship between de-trended and residual components of normalized monthly search volumes and monthly CPI for Pacific flying squid (Todarodes pacificus).

*
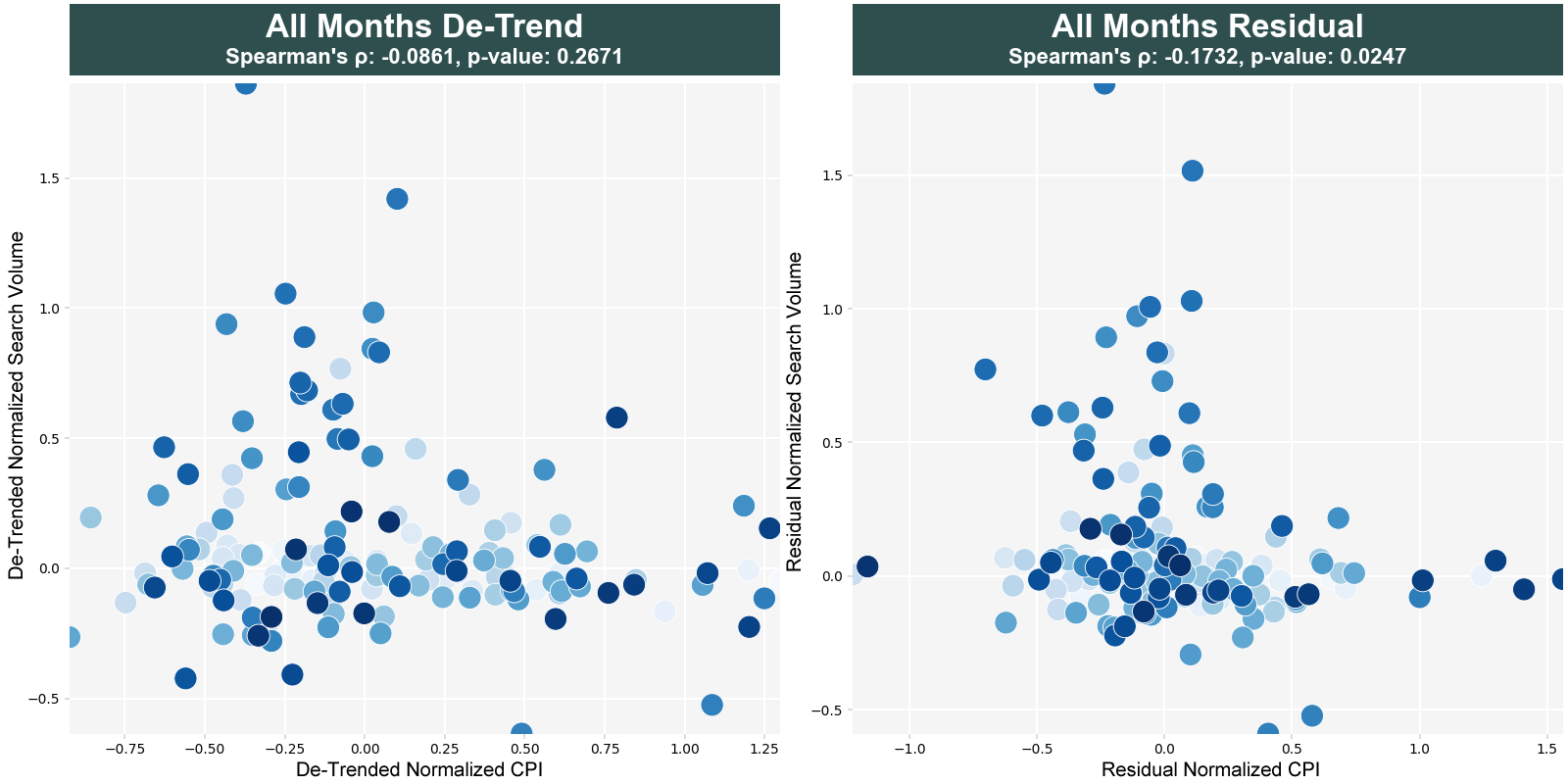
*

Figure S4.5. Relationship between de-trended and residual components of normalized monthly search volumes and monthly CPI for Mackerel (Scomber japonicus, Scomber aus-tralasicus).

*
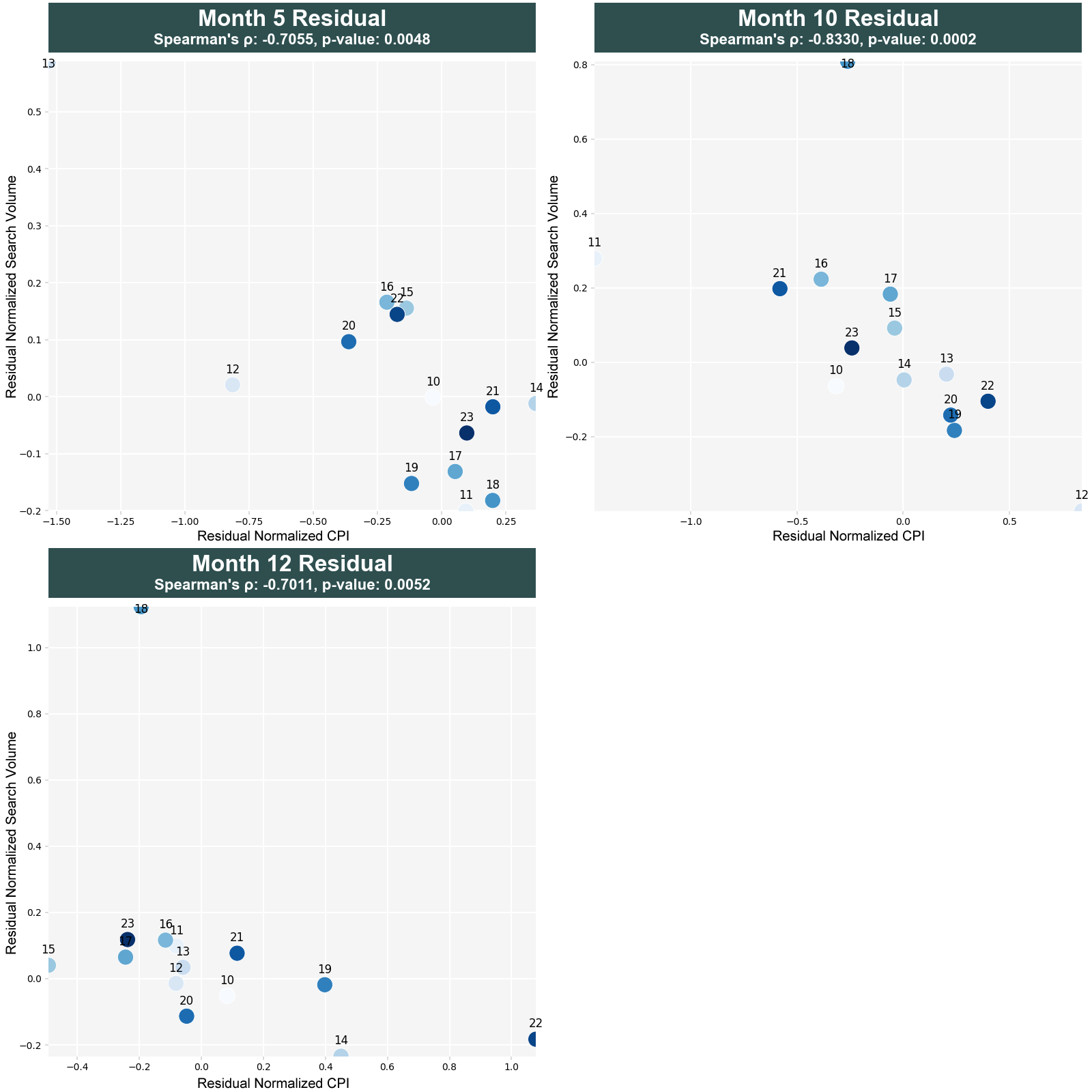
*

Figure S5.1. Relationship between residual components of normalized monthly search volumes and monthly CPI in March, October and December for Japanese sardine (Sardinops meknostictus).


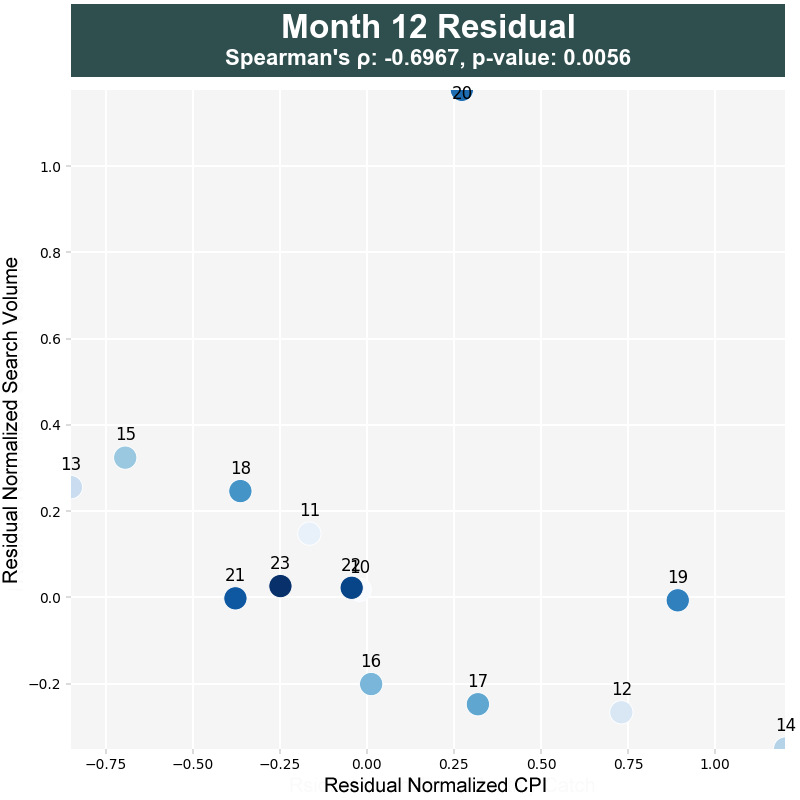


Figure S5.2. Relationship between residual components of normalized monthly search volumes and monthly CPI in December for Horse mackerel (Trachurus japonicus).

*
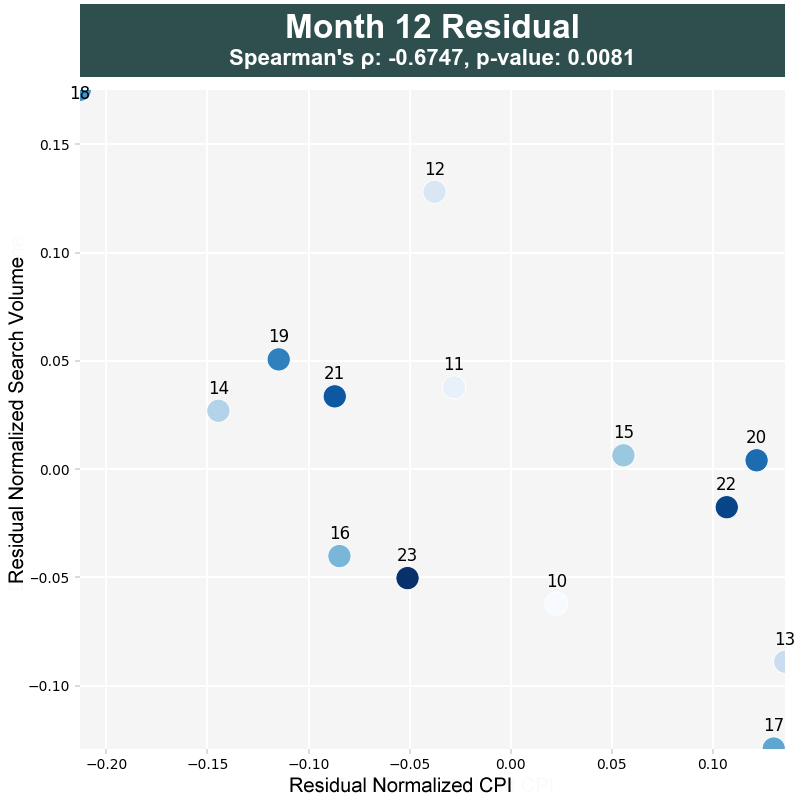
*

Figure S5.3. Relationship between residual components of normalized monthly search volumes and monthly CPI in December for Pacific saury (Cololabis Saira).

Figure. S6. Relationship between search volumes and log-transformed catch volumes across prefectures.

Purple dots represent northern prefectures, while yellow dots indicate southern prefec-tures. Each point is labeled with a prefecture number for identification. Results by species are shown below. All figures are in the same format.


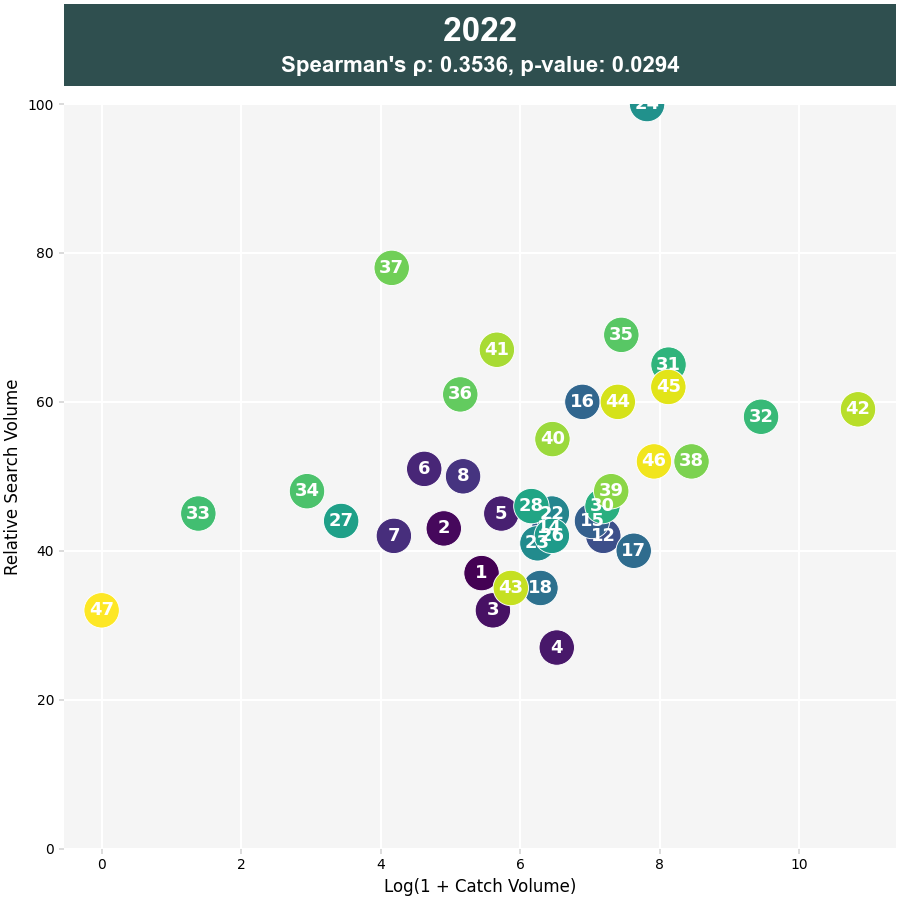


Figure S6.1. Relationship between search volumes and log-transformed catch volumes across prefectures in 2022 for Horse mackerel (Trachurus japonicus).


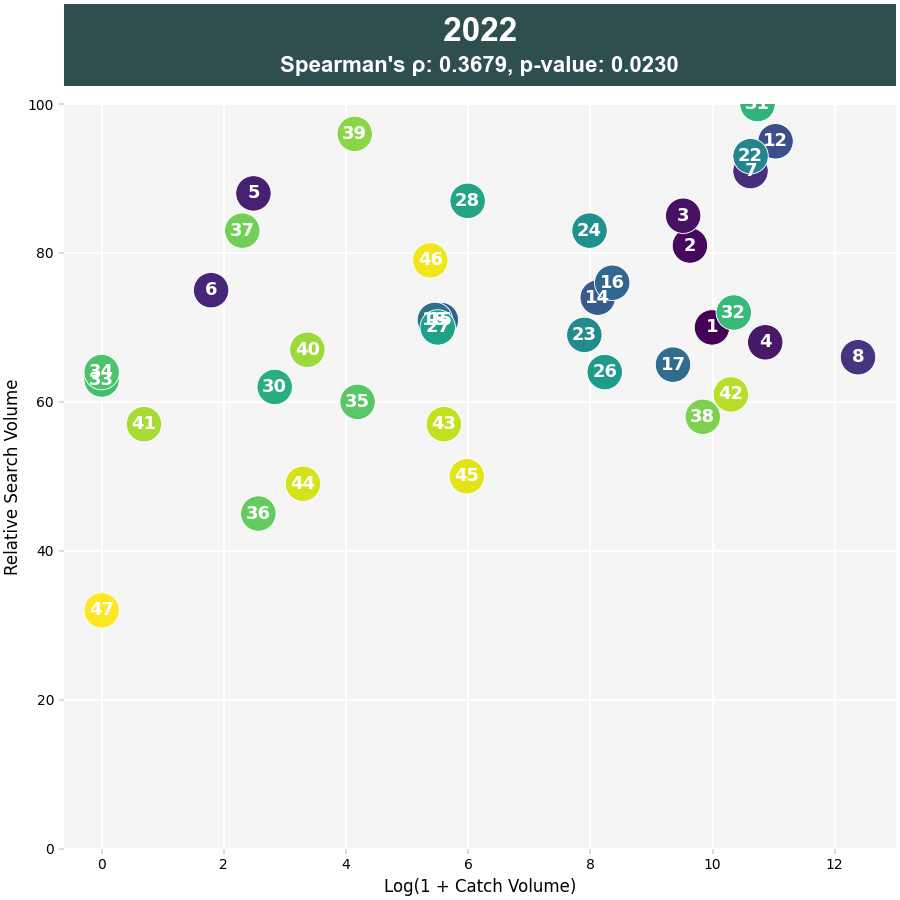


Figure S6.2. Relationship between search volumes and log-transformed catch volumes across prefectures in 2022 for Japanese sardine (Sardinops meknostictus).


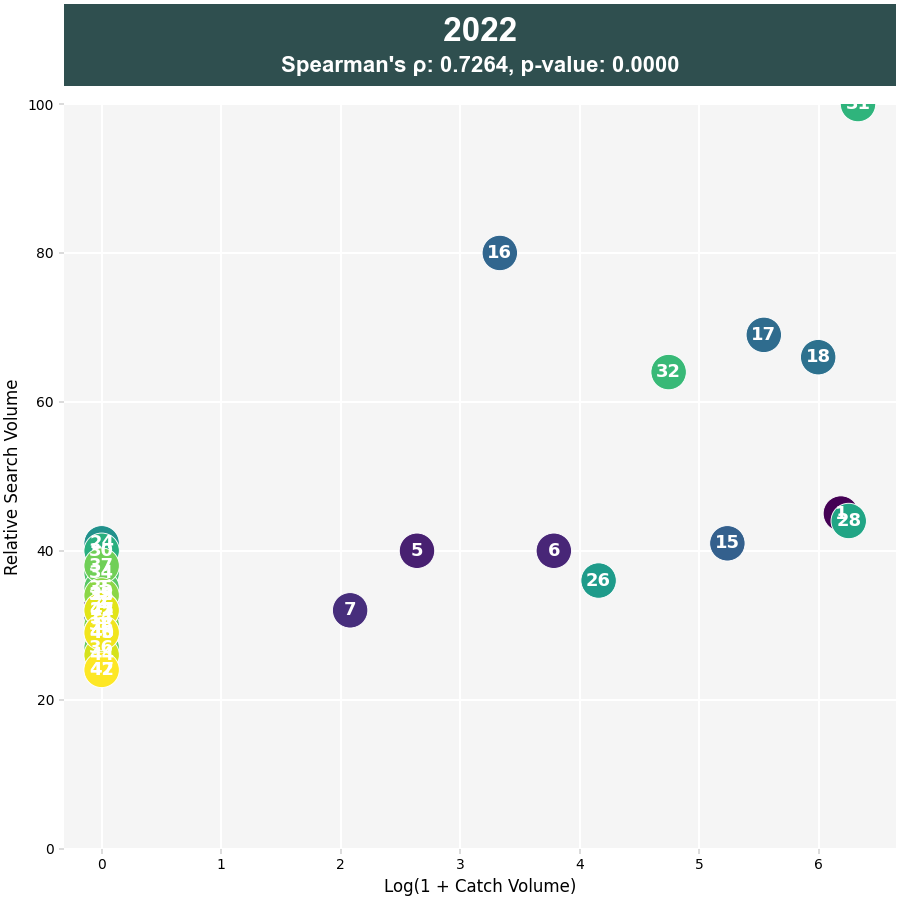


Figure S6.3. Relationship between search volumes and log-transformed catch volumes across prefectures in 2022 for Snow crab (Chionoecetes opilio).


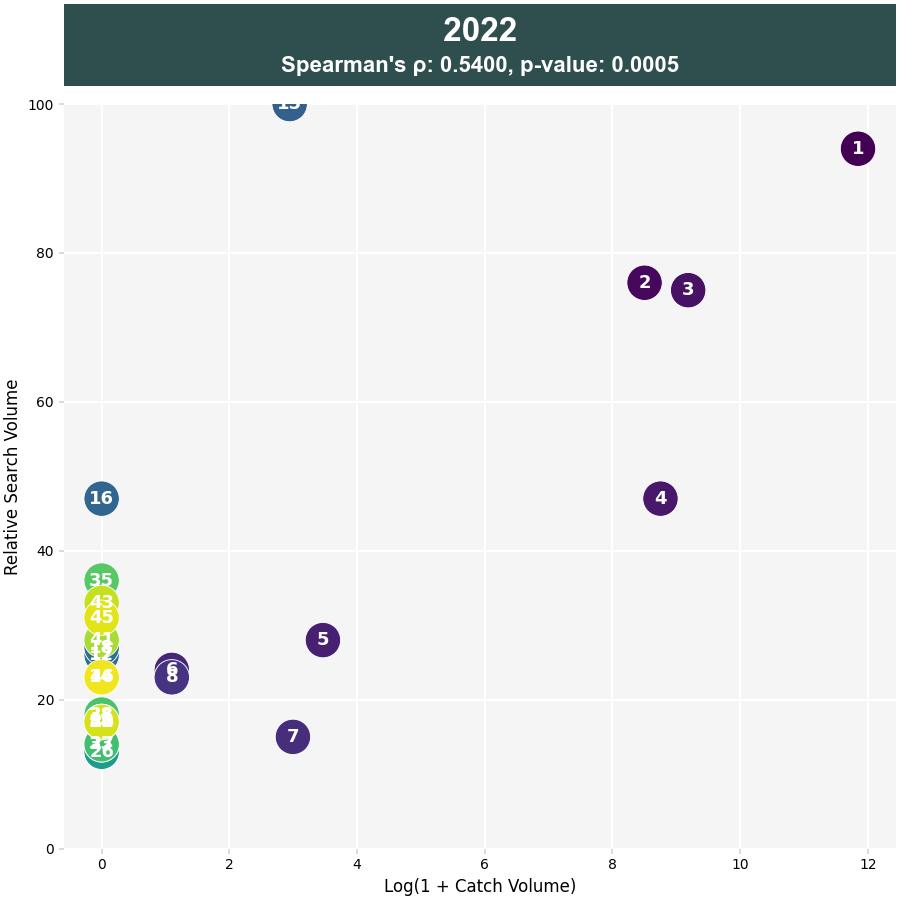


Figure S6.4. Relationship between search volumes and log-transformed catch volumes across prefectures in 2022 for Alaska pollack (Gadus chalcogrammus).


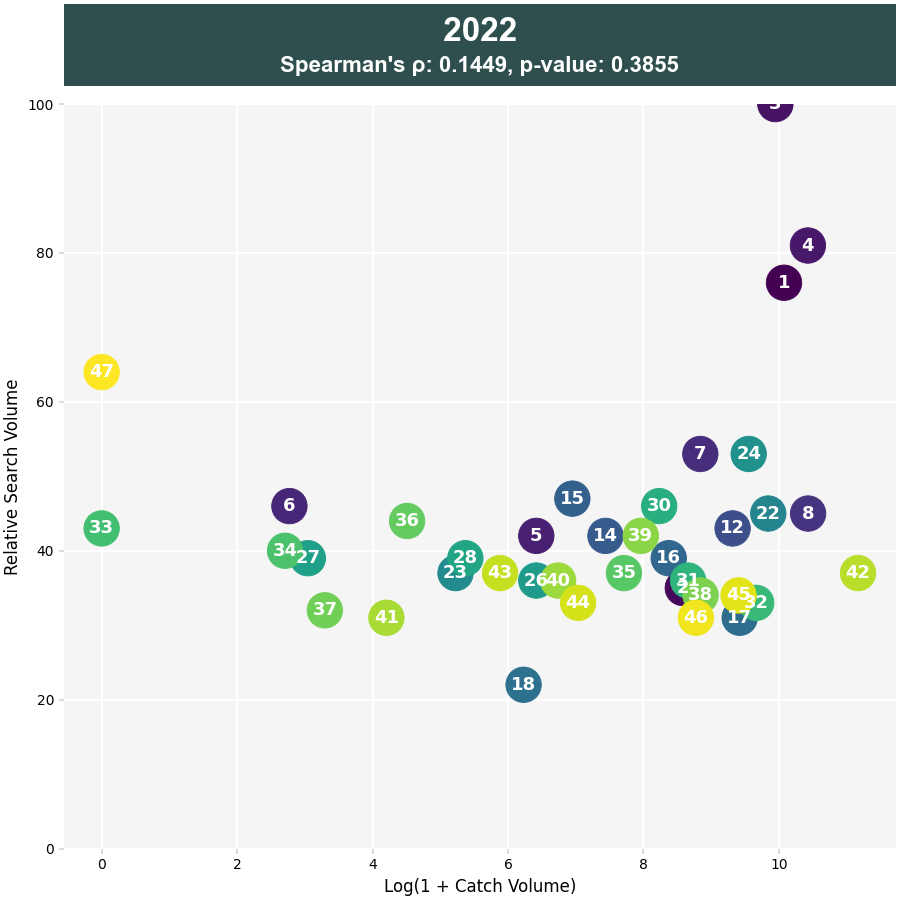


Figure S6.5. Relationship between search volumes and log-transformed catch volumes across prefectures in 2022 for Pacific saury (Cololabis Saira).


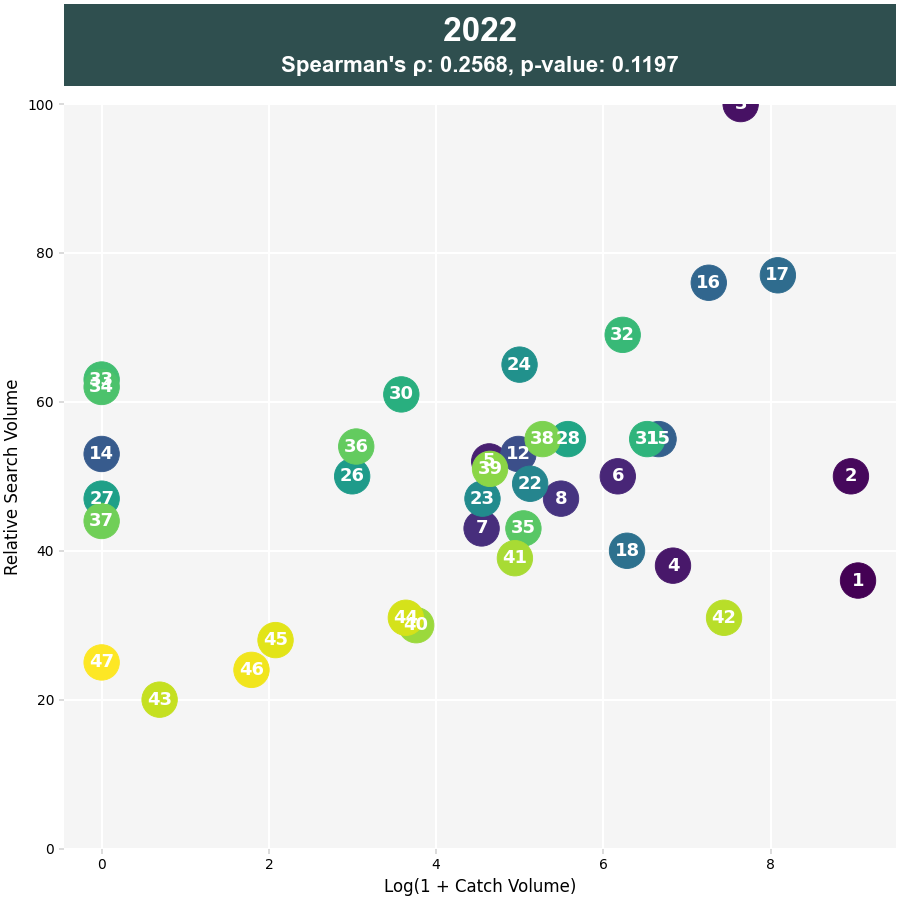


Figure S6.6. Relationship between search volumes and log-transformed catch volumes across prefectures in 2022 for Pacific flying squid (Todarodes pacificus).


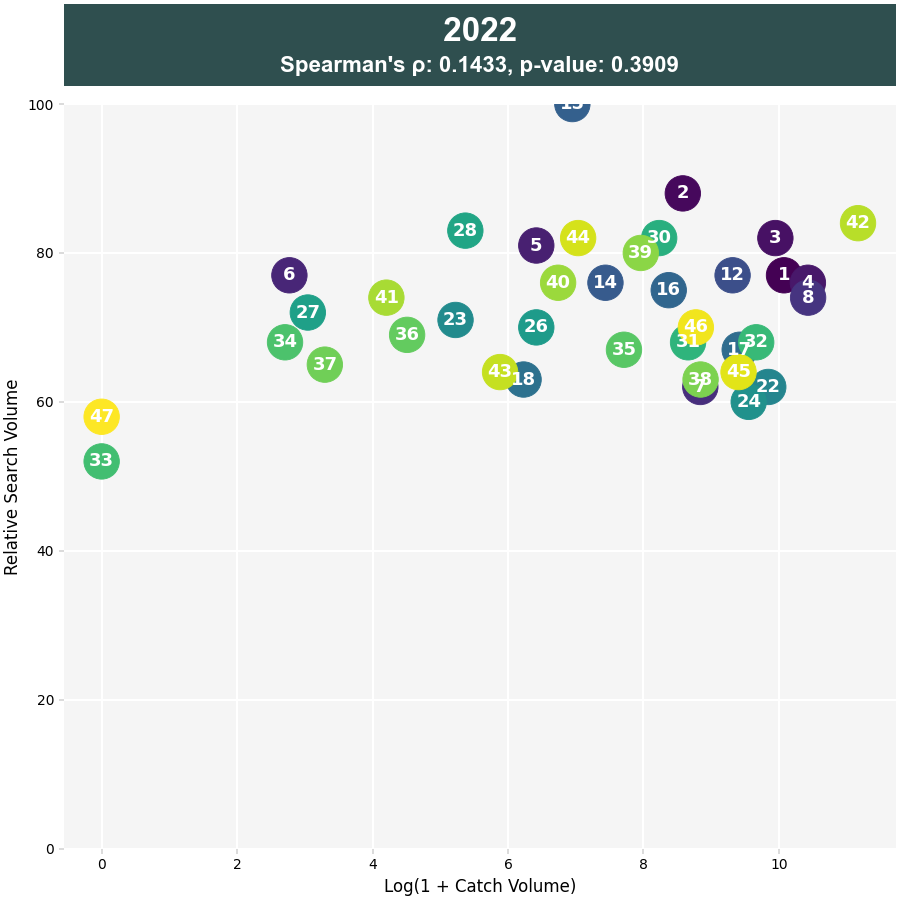


Figure S6.7. Relationship between search volumes and log-transformed catch volumes across prefectures in 2022 for Mackerel (Scomber japonicus, Scomber australasicus).
